# DNA interstrand cross-links induced by the major oxidative adenine lesion 7,8-dihydro-8-oxoadenine

**DOI:** 10.1038/s41467-021-22273-2

**Published:** 2021-03-26

**Authors:** Aaron L. Rozelle, Young Cheun, Caroline K. Vilas, Myong-Chul Koag, Seongmin Lee

**Affiliations:** 1 Division of Chemical Biology and Medicinal Chemistry, College of Pharmacy, The University of Texas at Austin, Austin, TX USA; 2 McKetta Department of Chemical Engineering, Cockrell School of Engineering, The University of Texas at Austin, Austin, TX USA; 3 Institute for Cellular and Molecular Biology, The University of Texas at Austin, Austin, TX USA

**Keywords:** DNA adducts, DNA

## Abstract

Oxidative damage to DNA generates 7,8-dihydro-8-oxoguanine (oxoG) and 7,8-dihydro-8-oxoadenine (oxoA) as two major lesions. Despite the comparable prevalence of these lesions, the biological effects of oxoA remain poorly characterized. Here we report the discovery of a class of DNA interstrand cross-links (ICLs) involving oxidized nucleobases. Under oxidative conditions, oxoA, but not oxoG, readily reacts with an opposite base to produce ICLs, highlighting a latent alkylating nature of oxoA. Reactive halogen species, one-electron oxidants, and the myeloperoxidase/H_2_O_2_/Cl^−^ system induce oxoA ICLs, suggesting that oxoA-mediated cross-links may arise endogenously. Nucleobase analog studies suggest C2-oxoA is covalently linked to N2-guanine and N3-adenine for the oxoA-G and oxoA-A ICLs, respectively. The oxoA ICLs presumably form via the oxidative activation of oxoA followed by the nucleophilic attack by an opposite base. Our findings provide insights into oxoA-mediated mutagenesis and contribute towards investigations of oxidative stress-induced ICLs and oxoA-based latent alkylating agents.

## Abstract (web summary)

7,8-dihydro-8-oxoguanine and 7,8-dihydro-8-oxoadenine (oxoA) are generated upon oxidative damage to DNA, but the biological effects of oxoA are not well known. Here, the authors report that only oxoA forms DNA interstrand crosslinks (ICLs) upon secondary oxidation and that these ICLs can be induced by reactive halogen species, one-electron oxidants and the myeloperoxidase/H_2_O_2_/Cl^-^ system.

## Introduction

**Fig. 1:**
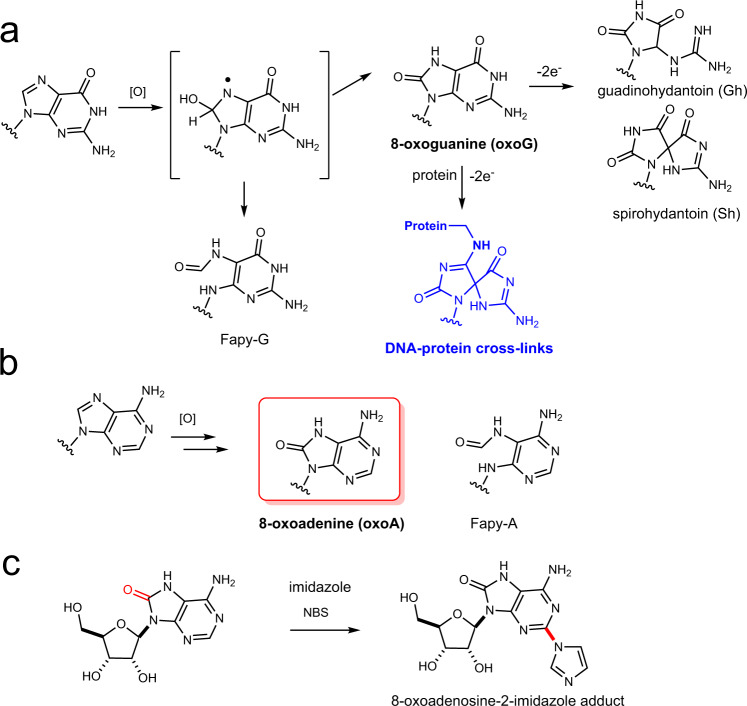
Representative 8-oxopurine lesions. **a** Formation of 8-oxoguanine and related lesions from oxidation of guanine. Note that oxidative activation of oxoG in the presence of proteins can produce DNA-protein cross-links. **b** Formation of 8-oxoadenine and Fapy-A from oxidation of adenine. **c** Formation of the oxoA-imidazole adduct from the reaction of oxoA with imidazole in the presence of *N*-bromosuccinimide. [O] an oxidation event. NBS *N*-bromosuccinimide, Fapy-G/A formaminopyrimidine guanine/adenine.

Oxidative damage to DNA is a ubiquitous process that involves reactive oxygen species (ROS)^1^. The levels of intracellular ROS increase under oxidative stress associated with metabolic strain, the inflammatory response, and cancer^2–4^. Due to the lower reduction potentials of purines, ROS preferentially attack purines in DNA to form the mutagenic 7,8-dihydro-8-oxoguanine (oxoG) and 7,8-dihydro-8-oxoadenine (oxoA) as two major oxidative adducts (Fig. 1)^5^, of which 10,000 lesions are produced per cell per day^6^. The rate of oxoA formation is found to be approximately one-tenth of oxoG, due to the higher redox potential of adenine residues over guanine^1,7^. However, there are certain conditions where the levels of oxoA have been comparable to that of oxoG. Specifically, the ratio of oxoA and oxoG modifications in larynx tumors and adjacent tissues is closer to 1:1^8^. The 8-oxopurines are more susceptible to oxidation than the undamaged purines because the redox potentials of oxoG (0.74 V) and oxoA (0.92 V) are significantly lower than those of guanine (1.29 V) and adenine (1.42 V)^5^. Indeed, oxoG readily undergoes further oxidation to generate the secondary oxidative lesions, spiroiminohydantoin (Sp) and 5-guanidinohydantoin (Gh)^9^. In addition, oxoG can react with lysine or tyrosine to produce protein–DNA cross-links under the influence of oxidants (Fig. 1a)^10–12^. Secondary lesions such as formamidopyrimidine (Fapy) lesions are also detected upon further oxidation of 8-oxopurines (Fig. 1b)^13,14^. Interestingly, oxoA ribonucleosides form stable adducts when treated with various nucleophiles (e.g., methanol, imidazole) under oxidative conditions^10^ (Fig. 1c), suggesting that oxoA within duplex DNA may be capable of forming DNA interstrand cross-links (ICLs) under similar conditions. While there are a wide variety of known secondary lesions of 8-oxopurines, the formation of ICLs induced by oxidized nucleobases is a rare occurrence. Some examples of intrastrand cross-links, which are covalently linked nucleobases on the same strand of DNA, generated by oxidizing species have been reported in the literature. These include cytosine-cytosine cross-links generated by 2-methyl-1,4-naphthoquinone, guanine-thymine cross-links formed by carbonate radical anions, and similar guanine-thymine cross-links formed by hydroxyl radicals^15–17^. Conversely, DNA ICLs generated by thymine radicals are the only example of oxidatively induced interstrand cross-linking in the literature to our knowledge^18^. The goal of the present work was to evaluate the capability of 8-oxopurines to form ICLs under oxidative conditions mimicking oxidative stress.

DNA ICLs are highly genotoxic lesions that occur when the complementary strands of duplex DNA become covalently linked^19^, which inhibits essential cellular functions such as replication and transcription^20^. The toxicity of ICLs toward dividing cells has led to widespread clinical usage of ICL-inducing agents (e.g., cisplatin, nitrogen mustards) in cancer treatment, and cellular resistance to these anti-cancer alkylating agents has been a primary driver of research regarding ICL formation, recognition, and repair^21^. In addition, the search for endogenous ICLs resulted in the identification of novel classes of ICLs including those induced by alcohol, unsaturated aldehydes, abasic sites, and colibactin^22–25^.

ICLs are of particular interest to the pathology of the rare genetic disorder, Fanconi anemia (FA), which is caused by mutations in a cluster of genes associated with replication-coupled ICL repair^26,27^. Interestingly, it has been shown that cell lines from patients suffering from FA are extremely sensitive to oxidative stress^28–31^. This observation led us to speculate that oxidative stress may promote the formation of ICLs from the abundant 8-oxopurines.

Here we report the discovery of a class of ICLs induced by secondary oxidation of the major oxidative adenine lesion oxoA. The cross-link formation was evaluated across a range of physiologically relevant oxidants including reactive halogen species (RXS), one-electron oxidant, and the myeloperoxidase (MPO)/hydrogen peroxide (H_2_O_2_)/Cl^−^ system. Distinct cross-links involving various nucleobases were characterized kinetically and mechanistically. These results suggest that the oxoA-mediated ICLs may be produced endogenously, particularly in cells experiencing oxidative stress. The oxoA ICLs do not involve abasic sites nor alkylating agents (e.g., unsaturated aldehydes, nitrogen mustards)^23,32,33^ and are induced by the oxidative activation of nucleobases.

## Results

### Modeling of oxoA-containing oligonucleotides for cross-linking studies

**Fig. 2:**
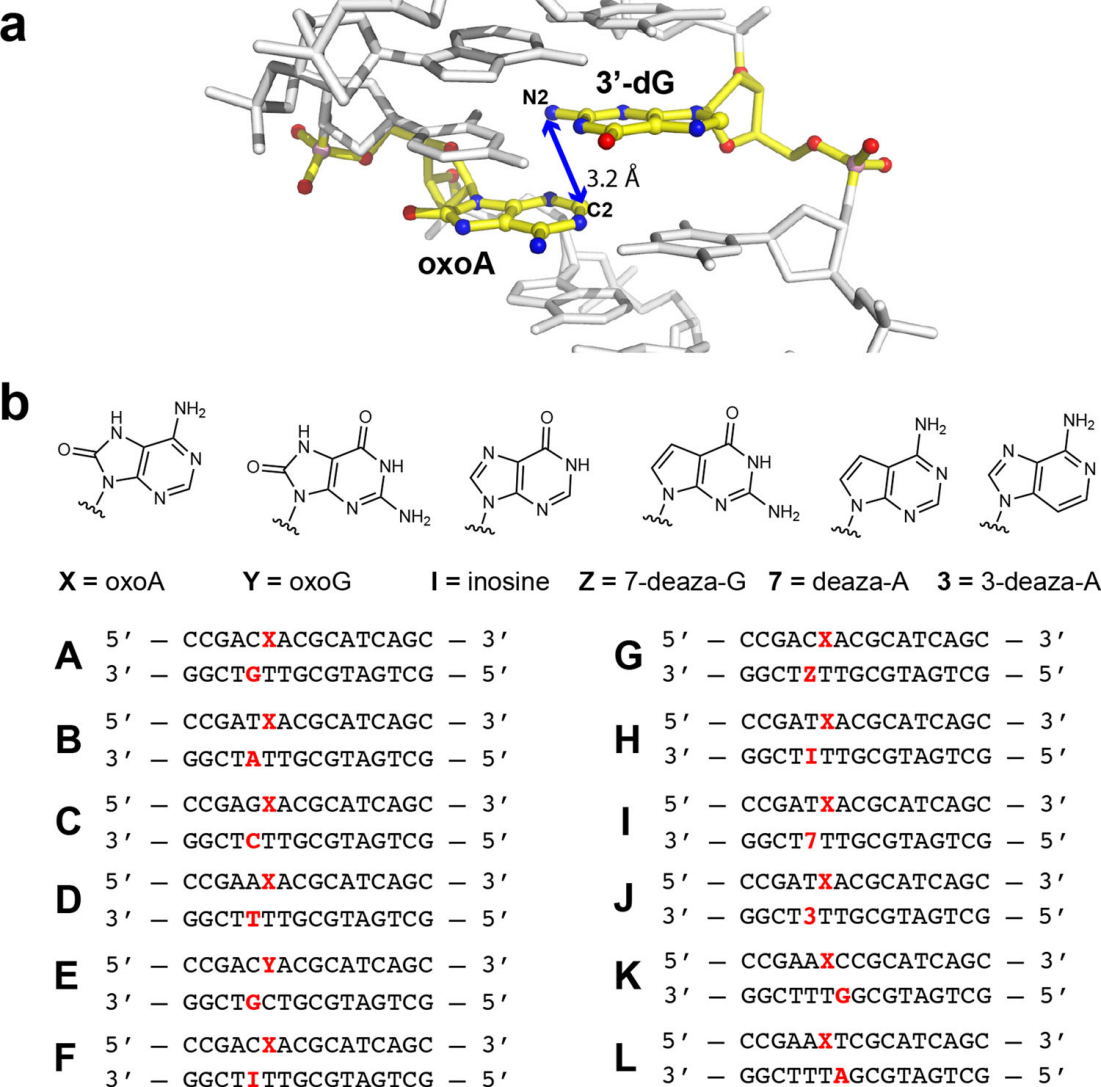
Oligonucleotide sequences used in this study. **a** Molecular model of duplex **A** showing the proximity of the N2 amine of the 3′-G residue and the C2 of oxoA in the 5′-C**X**/**G**T-5′ sequence (**X** = 8-oxoadenine [oxoA]; interstrand cross-linking [ICL] sites are underlined). The image was generated based on modification to PDB entry 1BNA [10.2210/pdb1BNA/pdb]. Distances between the C2 of oxoA and nucleophilic nitrogen in the 5′-CX/GT-5′, 5′-TX/AT-5′, and 5′-GX/CT-5′ sequences are 3.2 Å (N2 of dG), 4.6 Å (N6 of dA)/3.2 Å (N3 of dA), and 5.0 Å (N4 of dC), respectively. **b** Oligonucleotide sequences and structures of modified nucleobases used in this study to test ICL formation. The putative cross-linking sites are colored in bold red. X and Y represents oxoA and oxoG, respectively.

Modeling conducted in UCSF Chimera using a crystal structure of a short DNA duplex (PDB entry 1BNA)^34^ enabled our determination of DNA sequences that place the C2 of oxoA nearest the exocyclic amino groups of opposing guanine (G), adenine (A), and cytosine (C) residues (Fig. 2a). It should be noted that this modeling approach that prioritizes proximity was a starting point for our analyses; other factors such as orbital alignment, helical distortion, and bond angles likely play a greater role in influencing the reactivity of this system. Due to the nucleophilic nature of the exocyclic amines in nucleobases, we believed these contexts would be preferential for ICL formation^24,35^, and thus, oligonucleotides (ODNs) bearing single oxoA modifications were designed based on our modeling findings. Since thymine (T) does not possess an exocyclic amine that would act as a nucleophile, we anticipated that T would not cross-link with oxoA. This led to construction of duplexes **A**–**D** (Fig. 2b) with the general sequence 5’-MX/NT-5′, where X is oxoA; M is G, A, C, or T; N is the correct base pair of M; and the bases hypothesized to be involved in ICL formation are marked in bold.

### Specificity of 8-oxopurine-mediated formation of DNA interstrand cross-links

**Fig. 3:**
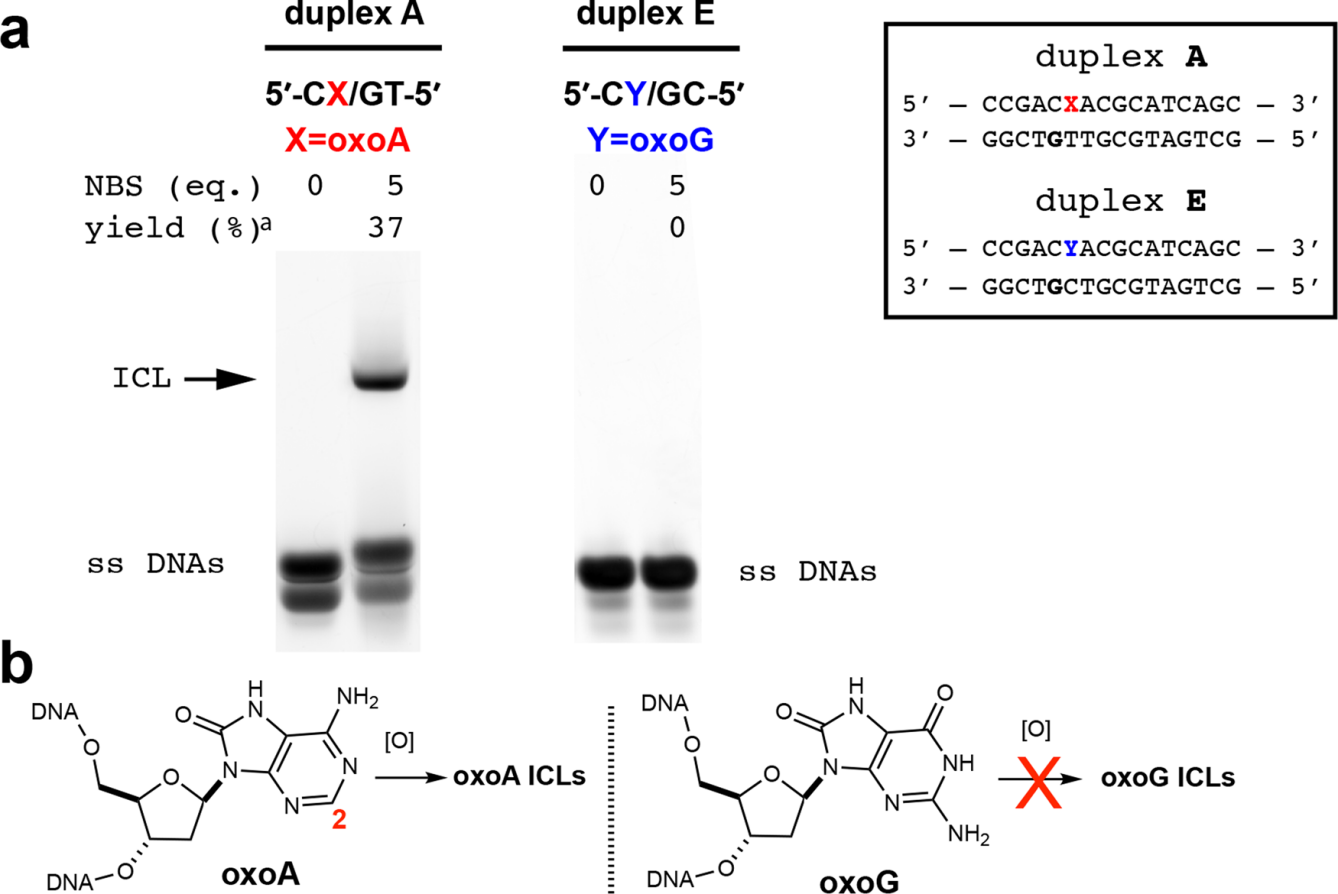
Specificity of 8-oxopurine-induced ICL formation. **a** Denaturing urea polyacrylamide gel electrophoresis analysis of NBS-catalyzed ICL reactions with duplexes **A** and **E** bearing 5′-CoxoA/GT-5′ and 5′-CoxoG/GT-5′ sequences, respectively. Duplex **A** produces a slow-migrating band, while duplex **E** does not produce ICL in the presence of NBS. Cross-linking yields were determined by dividing the percent band intensity of the cross-linked DNA band by the sum of the intensities of the unreacted complementary and oxoA strands (yield^a^). **b** Further oxidation of oxoA-containing duplex **A**, but not the oxoG-containing duplex **E**, produces ICL, suggesting the C2-H of 8-oxopurine is required for the oxidation-induced generation of ICLs. All yields and related statistics were derived from independent experiments and reported as the mean value ± SEM in the text based on three independent replicates. OxoA/G represents 8-oxoadenine/guanine, NBS *N*-bromosuccinimide, ICL interstrand cross-linking, [O] an oxidation event. Uncropped gel images may be found in the Supplementary information.

We first evaluated whether 8-oxopurine-containing duplex can form ICLs under oxidative conditions involving *N*-bromosuccinimide (NBS). To this end, we performed NBS-mediated oxidation of duplexes **A** and **E**, which contain 5′-C(oxoA)/GT-5′ and 5′-C(oxoG)/GC-5’, respectively. Treatment of duplex **A** with 5 molar equivalents of NBS (0.1 mM final concentration) produced a slow-migrating band in the expected region of ICL-bearing duplex (Fig. 3a). The MALDI-TOF-MS data for the cross-linked duplex **A** were consistent with the cross-link formation, giving an observed m/z of 9778.028 for the cross-linked duplex **A** (expected m/z 9778.446; Supplementary Fig. 5). Control experiments without NBS (Fig. 3a), with undamaged base (Supplementary Fig. 1), or with single-stranded ODNs (Supplementary Figs. 3 and 4) did not generate a slow-migrating band, indicating that the cross-linking occurs via further oxidation of oxoA-containing duplex DNA. To determine whether the C2-H of the 8-oxopurine is required for 8-oxopurine-mediated ICL formation, we conducted NBS oxidation of duplexes **E** bearing oxoG (no C2-H) in place of oxoA (Fig. 3a). Unlike the oxoA-containing duplex **A**, the oxoG-containing duplexes **E** did not produce a slow-migrating band (Fig. 3a), showing that oxoG does not induce ICL formation in the presence of NBS. Overall, these results indicate that an 8-oxopurine unsubstituted at the C2 position is required for these lesions to initiate cross-linking (Fig. 3b), highlighting the specificity of oxoA in the oxidation-induced formation of ICLs.

### NBS-induced formation of cross-links between oxoA and nucleobases

Having discovered that oxoA can induce cross-linking with an opposite guanine under the influence of NBS, we next explored whether the major oxidative adenine lesion can form covalent bonds with other nucleobases. To this end, we carried out NBS oxidation of duplexes **A**, **B**, **C**, and **D**, which contain 5′-C(oxoA)/GT-5′, 5′-ToxoA/AT-5′, 5′-G(oxoA)/CT-5′, and 5′-A(oxoA)/TT-5′, respectively (Supplementary Fig. 7). The cross-linked duplex **A**, which possesses the putative covalent link between oxoA and G, was observed at yields of 37.3 ± 0.4% based on polyacrylamide gel electrophoresis (PAGE) analysis of stained, denaturing urea gels and 23.3 ± 2.0% based on quantification of PAGE-purified, gel-extracted cross-linked DNA (Supplementary Fig. 7, lanes 1–3). Gel extraction purification and quantification of these cross-linking reactions was performed to ensure that reported ICL yields were accurate. SYBR Gold nucleic acid staining was used to quantify ICL yields by densitometry; however, this gel stain binds to ssDNA and double-stranded DNA (dsDNA) with differing efficiencies. Because the bands containing cross-linked DNA may not be entirely ssDNAs, this staining method may overstate the observed ICL yield. The NBS oxidation of duplex **B** containing 5′-ToxoA/AT-5′ gave rise to slow-migrating bands in the expected region of the cross-linked duplex (Supplementary Fig. 7, lanes 4–6). The MALDI-TOF-MS data for the cross-linked duplex **B** were consistent with the cross-link formation, yielding an observed m/z of 9776.353 for the cross-linked duplex **B** (expected m/z 9778.446; Supplementary Fig. 6). The cross-linked duplex **B**, which contains the putative oxoA-A adduct, was produced with a yield of 28.4 ± 0.5% based on PAGE analysis of stained, denaturing urea gels, while the yield from gel extraction and purification of the cross-linked duplex **B** was determined to be 15.6 ± 1.5% duplexes (Supplementary Fig. 7, lanes 4–6). Despite the same length and the similar ODN sequence of duplexes **A** and **B**, the oxoA-G ICL travels faster than the oxoA-A ICL during electrophoresis (Supplementary Fig. 7, lanes 3 and 7), suggesting the three-dimensional structures of the oxoA-G and oxoA-A ICLs differ. In addition to duplexes **A** and **B**, cross-link formation was evaluated for duplexes **C** and **D**, which contain 5′-G(oxoA)/CT-5′ and 5′-A(oxoA)/TT-5′, respectively. The ICL yields (~3% based on PAGE analysis) for duplex **C** were lower than those for duplexes **A** and **B** (Supplementary Fig. 7, lanes 7–9), indicating the cytosine in the 5′-G(oxoA)/CT-5′ context acts as a poor nucleophile for the oxidation-induced ICL formation. Interestingly, duplex **D** bearing a thymine opposite oxoA yielded slow-migrating bands while the yields were lower (8.9 ± 0.4% based on PAGE analysis) (Supplementary Fig. 7, lanes 10–12). The characterization of the oxoA-C and oxoA-T ICLs was difficult due to their low reaction yields. Therefore, for the remaining oxoA ICL studies, we focused our analyses on the oxoA-G and oxoA-A ICLs unless otherwise stated. Together, these results demonstrate that oxoA can form covalent bonds with various nucleobases under oxidative conditions.

### The effect of oxidants on the formation of the oxoA-G and oxoA-A ICLs

**Fig. 4:**
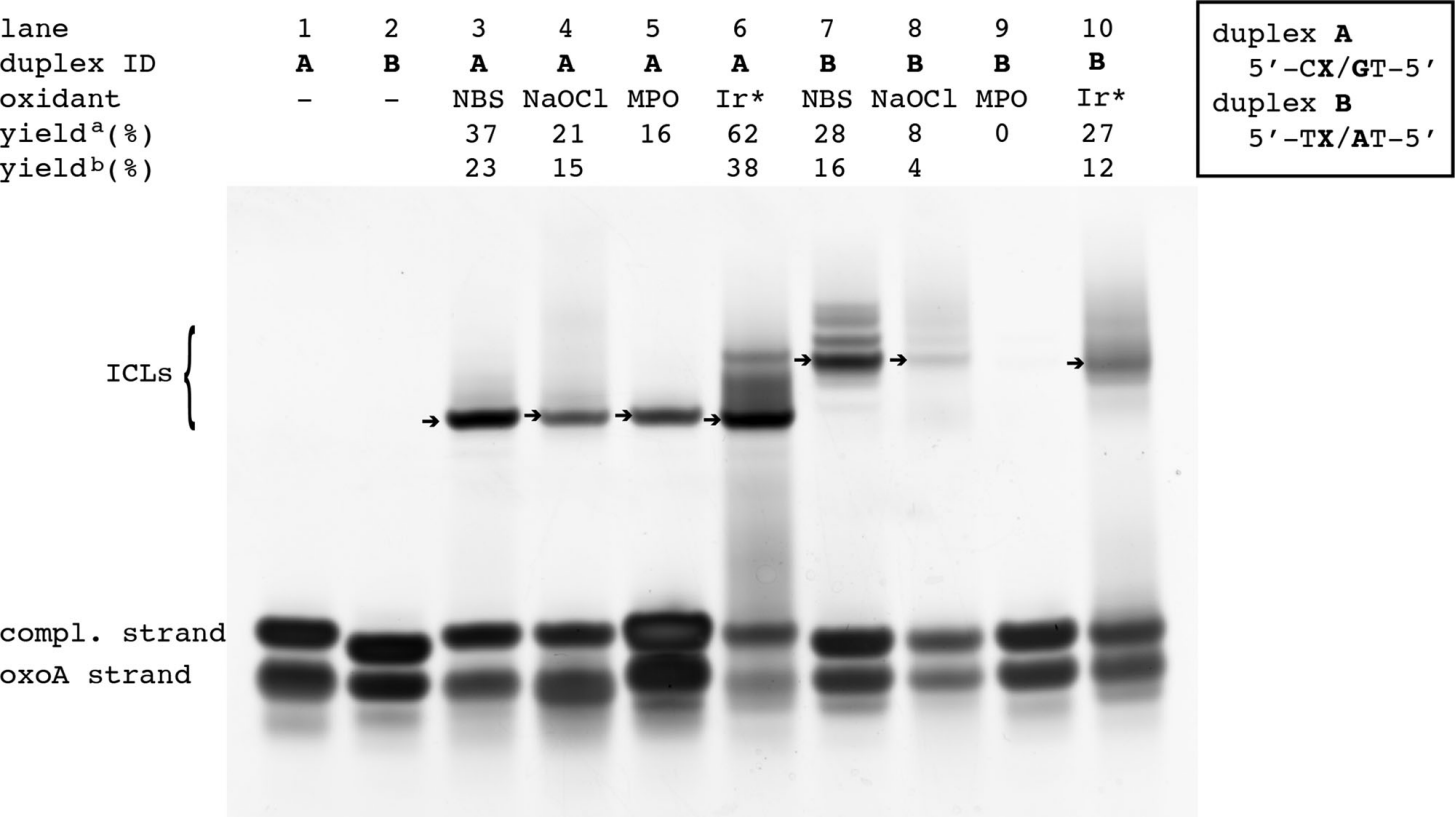
Effect of oxidants on the formation of the oxoA-G and oxoA-A cross-links. Denaturing PAGE analyses of oxoA ICL formation in the presence of NBS, NaOCl, MPO, or Na_2_IrCl_6_ (Ir*) are shown. Duplexes **A** and **B** contain 5′-CoxoA/GT-5′ and 5′-ToxoA/AT-5′sequence, respectively, where nucleophilic bases are underlined. Slow-migrating bands represent the oxoA ICLs, while the fast-migrating bands represent unreacted single-stranded DNA. The major oxoA ICLs from individual reactions are indicated as arrows and their ICL yields are shown. Cross-linking yields were determined by dividing the percent band intensity of the cross-linked DNA band by the sum of the intensities of the unreacted complementary and oxoA strands (yield^a^) or by quantification of PAGE-purified, gel-extracted cross-link DNA (yield^b^). Yields were calculated from three independent experiments. All yields and related statistics were derived from independent experiments and reported as the mean value ± SEM in the text based on three independent replicates. OxoA represents 8-oxoadenine and is defined as X in the figure. NBS *N*-bromosuccinimide, MPO myeloperoxidase, ICL interstrand cross-linking.

To evaluate the impact of oxidants on the formation of the oxoA ICLs, we performed cross-linking experiments by incubating various oxidants with the DNA duplexes **A** and **B**, which contain 5′-C(oxoA)/GT-5′ and 5′-T(oxoA)/AT-5′, respectively (Fig. 4). To assess whether oxoA-mediated cross-linking occurs under physiologically relevant conditions, we screened for cross-link formation using sodium hypochlorite (NaOCl). In aqueous solution, NaOCl is in equilibrium with hypochlorous acid (HOCl), a RXS produced in the human inflammatory response in order to kill pathogens^36,37^. Treatment of duplexes **A** and **B** with NaOCl (50 total molar equivalents, 1 mM final concentration) at 37 °C for 1.25 h resulted in the formation of the oxoA-G ICL (21.2 ± 1.9 %, Fig. 4, lane 4) and the oxoA-A ICL (8.2 ± 0.9 %, Fig. 4, lane 8), respectively. The yields for both ICLs based on quantification of the purified, cross-linked duplexes were 14.7 ± 1.7% and 4.2 ± 0.2%, respectively.

During the human inflammatory response, MPO utilizes H_2_O_2_ and chloride anions to generate HOCl. We evaluated whether the oxoA ICL forms under oxidation conditions mimicking the MPO-H_2_O_2_-halide system in chronic inflammation (Fig. 4, lanes 5 and 9, and Supplementary Fig. 8). These reactions were carried out by incubating duplex DNA (20 μM) with 250 μM H_2_O_2_, 100 mM NaCl, and 50 nM MPO. The MPO-catalyzed reaction of duplex **A** produced the oxoA-G ICL at a yield of 16.4 ± 1.0% (Fig. 4, lane 5), while that of duplex **B** resulted in no significant ICL formation (Fig. 4, lane 9). No gel-extraction quantification was performed for this set of reactions due to the cost of MPO required to carry out the reactions at scale. The formation of the oxoA ICLs in the presence of NaOCl and the MPO-H_2_O_2_-halide system suggests that oxoA-mediated ICL formation may occur in a cellular environment, particularly under oxidative stress.

RXS such as HOCl and NBS are two-electron oxidants and damage DNA in a discrete way compared with other oxidants such as ROS, which are commonly produced in the cellular environment^7,37,38^. To examine whether different oxidation mechanisms can induce oxoA-mediated ICL formation, we treated duplexes **A** and **B** with a one-electron oxidant, sodium hexachloroiridate (IV) (Na_2_IrCl_6_), and screened for cross-link formation (Fig. 4, lanes 6 and 10). Incubation of duplex **A** with Na_2_IrCl_6_ (200 total molar equivalents, 4 mM final concentration) generated the oxoA-G ICL with a yield of 62.1 ± 1.1% (lane 6), while the reaction of duplex **B** generated the oxoA-A ICL with a yield of 26.5 ± 1.0% (lane 10) based on PAGE analysis stained, denaturing urea gels. Quantification of the PAGE-purified, gel-extracted cross-linked duplexes revealed ICL yields of 38.2 ± 3.1% and 12.3 ± 1.0% for duplex **A** and **B**, respectively. The high reactivity of oxoA in favor of cross-link formation in the presence of this one-electron oxidant suggests that oxoA-mediated ICL formation may occur under general oxidative conditions.

### Identifying the participating moieties in the oxoA-G cross-linking reactions

It has been reported that the reaction of 8-oxoadenosine iminoquinone with nucleophiles (e.g., methanol, imidazole) occurs at the C2 of 8-oxoadenosine^13^. It is likely the oxoA-mediated ICL formation also occurs at the C2 position of 8-oxoadenine. Studies of abasic site-induced ICLs show that the exocyclic amines of purines (e.g., dA-N6, dG-N2) participate in the cross-link formation^24,35,39^. Together, these observations suggest that it is possible that the oxoA cross-linking reaction involves the exocyclic amines of the nucleophilic purines with the C2 position of oxoA. However, the endocyclic nitrogens of dG (N7) and dA (N3, N7), which are the predominant targets of DNA alkylating agents, could also partake in the oxoA ICL formation due to their high nucleophilicity^40–42^.

**Fig. 5:**
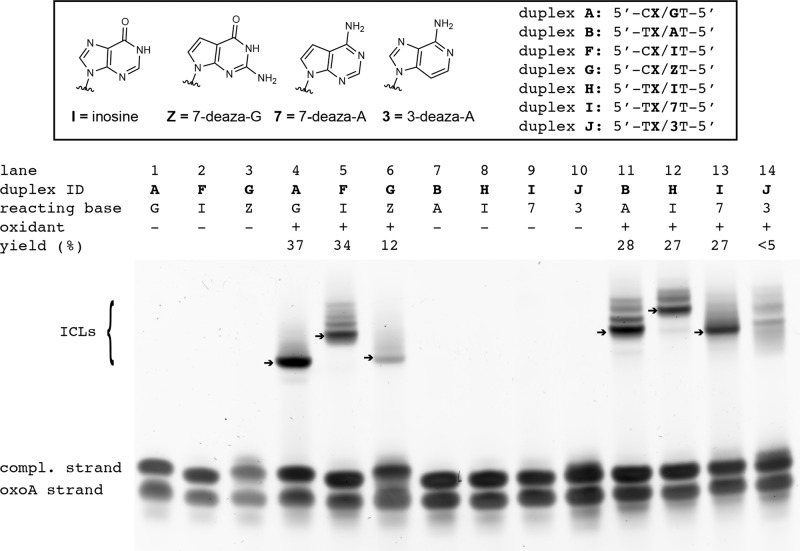
Denaturing urea PAGE analysis of NBS-catalyzed ICL reactions with base-modified DNA duplexes to identify functional groups involved in oxoA-mediated ICL reactions. Duplexes contain 5′-CoxoA/GT-5′ (duplex **A**), 5′-ToxoA/AT-5′ (**B**), 5′-CoxoA/IT-5′ (**F**), 5′-CoxoA/ZT-5′ (**G**), 5′-ToxoA/IT-5′ (**H**), 5′-ToxoA/7T-5′ (**I**), and 5′-ToxoA/3T-5′ (**J**) sequences. The major oxoA ICLs from individual reactions are indicated as arrows and their ICL yields are shown. Cross-linking yields were determined by dividing the percent band intensity of the cross-linked DNA band by the sum of the intensities of the unreacted complementary and oxoA strands. Yields are averages of three independent determinations. All yields and related statistics were derived from independent experiments and reported as the mean value ± SEM in the text based on three independent replicates. OxoA represents 8-oxoadenine and is defined as X in the figure. NBS *N*-bromosuccinimide, ICL interstrand cross-linking.

To determine the reactive moieties directly involved in the formation of the oxoA ICL, we performed cross-linking screens with duplexes **F**–**J**, which contained various modified bases in place of the canonical bases (e.g., A, G) believed to be involved in ICL formation (Figs. 2b and 5). Duplexes **F–J** possess nucleobases such as hypoxanthine (HX), 7-deazaguanine, 7-deazaadenine, or 3-deazaadenine opposite oxoA (Figs. 2b and 5). First, we evaluated NBS oxidation of duplex **F** containing inosine (I) (5′-C(oxoA)/IT-5′, Fig. 5, lane 5): Inosine lacks the N2 amino group and thus would preclude the cross-linking between oxoA and the N2 of a purine. The NBS treatment of duplex **F** gave the oxoA-I ICL with a good cross-linking yield (34.1 ± 0.7%). As inosine lacks the N2 amino group, the oxoA-I cross-linking reaction would involve the N7 and/or N3 of inosine. Interestingly, the oxoA-I ICL migrates slower than the oxoA-G ICL (*R*_*f*_ = 0.38 vs. 0.29 for the oxoA-G and oxoA-I ICLs, respectively), suggesting the three-dimensional structure of the oxoA-I ICL differs considerably from that of the oxoA-G ICL.

We also conducted NBS oxidation of duplex **G** containing 7-deazaguanine (Z), which contains a C7-H in place of the N7 of guanine (Fig. 5, lane 6). The 7-deaza modification precludes the participation of the purine N7 in oxoA ICL formation. Upon treatment with NBS, duplex **G** gave rise to the oxoA-Z ICL with a 11.8 ± 0.7% yield. The reduced cross-linking yield might be caused by the replacement of the electron-rich N7 with a C7 atom. Unlike the oxoA-I ICL, the oxoA-Z ICL migrated at a similar *R*_*f*_ to the oxoA-G ICL (Fig. 5, lanes 4 and 6), suggesting that the three-dimensional structures of both ICLs would be alike. The similar migration pattern of the oxoA-Z ICL as that of the oxoA-G ICL suggests that the N2 or N3 positions of guanine mainly engage in the oxoA-G ICL formation.

**Fig. 6:**
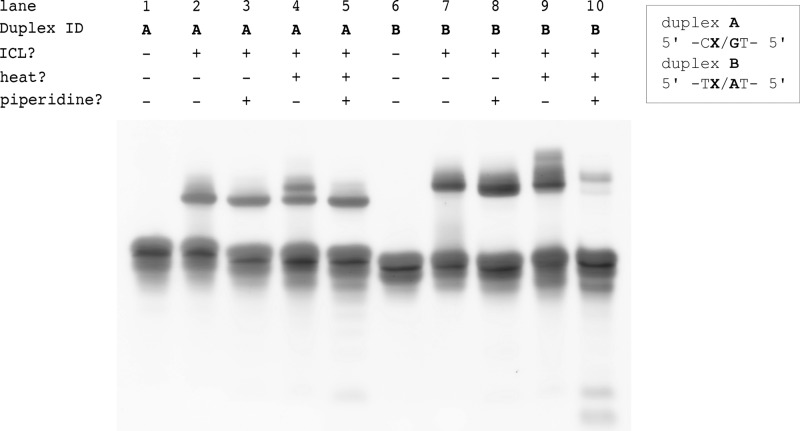
Denaturing urea PAGE analysis of cross-linked duplexes A and B treated with various combinations of heat and/or piperidine. Duplexes **A** and **B** contain 5′-CoxoA/GT-5′ and 5′-ToxoA/AT-5′sequence, respectively, where nucleophilic bases are underlined. The duplex **A** and **B** cross-linking reactions were heated for 30 min at 90 °C in the presence/absence of piperidine. The concentrated samples were resuspended in the formamide loading buffer and subjected to PAGE analysis. Duplicate reactions were performed to ensure accurate reporting of data. OxoA represents 8-oxoadenine and is defined as X in the figure. ICL interstrand cross-linking.

**Fig. 7:**
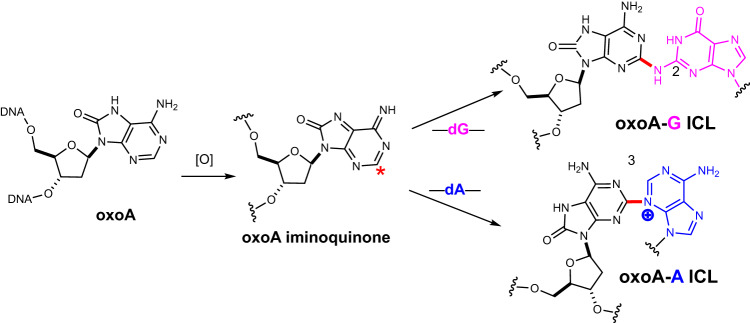
Proposed structures and mechanism for the oxoA-G and oxoA-A ICLs. Under oxidative conditions, 8-oxoadenine (oxoA) can be transformed into a purine iminoquinone intermediate, which can react with the exocyclic N2 of guanine residues or the endocyclic N3 of adenine residues to form interstrand cross-links (ICL). The red asterisk represents the electrophilic position of the iminoquinone intermediate generated upon oxidation of oxoA. Note that the oxoA-A ICL would be unstable due to the presence of positively charged adenine N3 alkyl group, which can promote degradation of the cross-link adducts.

To characterize the oxoA-G cross-links, we treated the duplex **A** cross-links with piperidine (Fig. 6, lanes 1–5). Piperidine treatment of N7 or N3 alkylated DNA, where a formal positive charge is present on the nitrogen, has been shown to promote single-strand breaks in duplex DNA and is often used to identify purine N7/N3-alkylation products in DNA studies^43^. In contrast, guanine N2- and adenine N6-alkylation adducts do not produce strand breaks upon piperidine treatment due to their chemical stability. Treatment of the cross-linked duplex **A** (oxoA-G ICL) with hot piperidine (90 °C, 30 min) resulted in no observable changes in cross-linking yield, indicating the oxoA-G ICL may contain a chemically stable linkage between the C2 of oxoA and the N2 of G (Fig. 7).

### Identifying the participating moieties in the oxoA-A cross-linking reactions

In addition to the oxoA-G ICL, we assessed the chemical structure of the oxoA-A ICL by conducting cross-linking screens with duplexes **H**, **I**, and **J**, which also contain modified bases. The nucleophilic adenine in duplex **B** was replaced with inosine (duplex **H**), 7-deazaadenine (duplex **I**), or 3-deazaadenine (duplex **J**). The NBS treatment of duplex **H** bearing inosine produced the oxoA-inosine ICL with a 26.7 ± 1.5% yield (Fig. 5, lane 12), which is comparable to the oxoA-A ICL formation (28.4 ± 0.5%). Note that both duplexes **F** and **H** contain inosine but their sequences vary (5′-CX/IT-5′ vs. 5′-TX/IT-5′, respectively). The migration of the oxoA-inosine ICL from duplex **H** was slower than the oxoA-A ICL (band *R*_*f*_ value: 0.22 vs. 0.29), suggesting that the structure of the oxoA-inosine ICL differs from that of the oxoA-A ICL structure. As in the reaction involving duplex **F** (5′-CX/IT-5′), replacement of adenine with inosine in duplex **H** removes the possibility of the participation of the exocyclic N6 amine in cross-linking; however, inosine is still capable of forming adducts at the N1, N3, and N7 positions.

The further oxidation of the 7-deazaadenine-modified duplex **I** resulted in the oxoA-7-deazaadenine ICL with a decent cross-linking yield (26.8 ± 0.4%) and a near identical band migration pattern (*R*_f_ = 0.27, Fig. 5, lane 13) as that of the oxoA-A ICL (lane 11). In contrast, the use of duplex **J** containing 3-deazaadenine did not produce major ICL adducts, resulting in a low (<5%) ICL yield (Fig. 5, lane 14). As the modified bases of duplexes **I** and **J** lack the endocyclic N7 and N3, respectively, these results suggest that the N3 position of adenine, rather than N6 or N7, is the major participant in the oxoA-A ICL formation (Fig. 7).

As with the oxoA-G cross-link, the oxoA-A ICL appears to be quite stable at high temperatures (90 °C; Fig. 6, lane 9). However, piperidine workup of the duplex **B** cross-linking reaction resulted in extensive degradation of the major oxoA-A cross-link adduct (Fig. 6, lane 10). The labile nature of the major oxoA-A ICL product suggests the exocyclic N6 of adenine may not be the main participant in the cross-linking reaction, as the oxoA-A ICL involving the N6 of adenine is expected to be stable under these caustic conditions. Interestingly, the slower migrating cross-linked band did not greatly degrade upon piperidine treatment (Fig. 6, lane 10), suggesting that this minor band may represent a unique cross-link. Based on these observations, we conclude that the major form of the oxoA-A ICLs would possess a linkage between the C2 of oxoA and the endocyclic N3 of dA. The oxoA-A ICL with such a linkage would have a formal positive charge on the oxoA-A adduct, thereby making it susceptible to a piperidine workup. Furthermore, the piperidine lability of the major oxoA-A ICL band suggests that the cross-link may be reversible in nature, which implies that the observed yields reported here may be understated.

### Identifying secondary reactions occurring during oxoA cross-linking

**Fig. 8:**
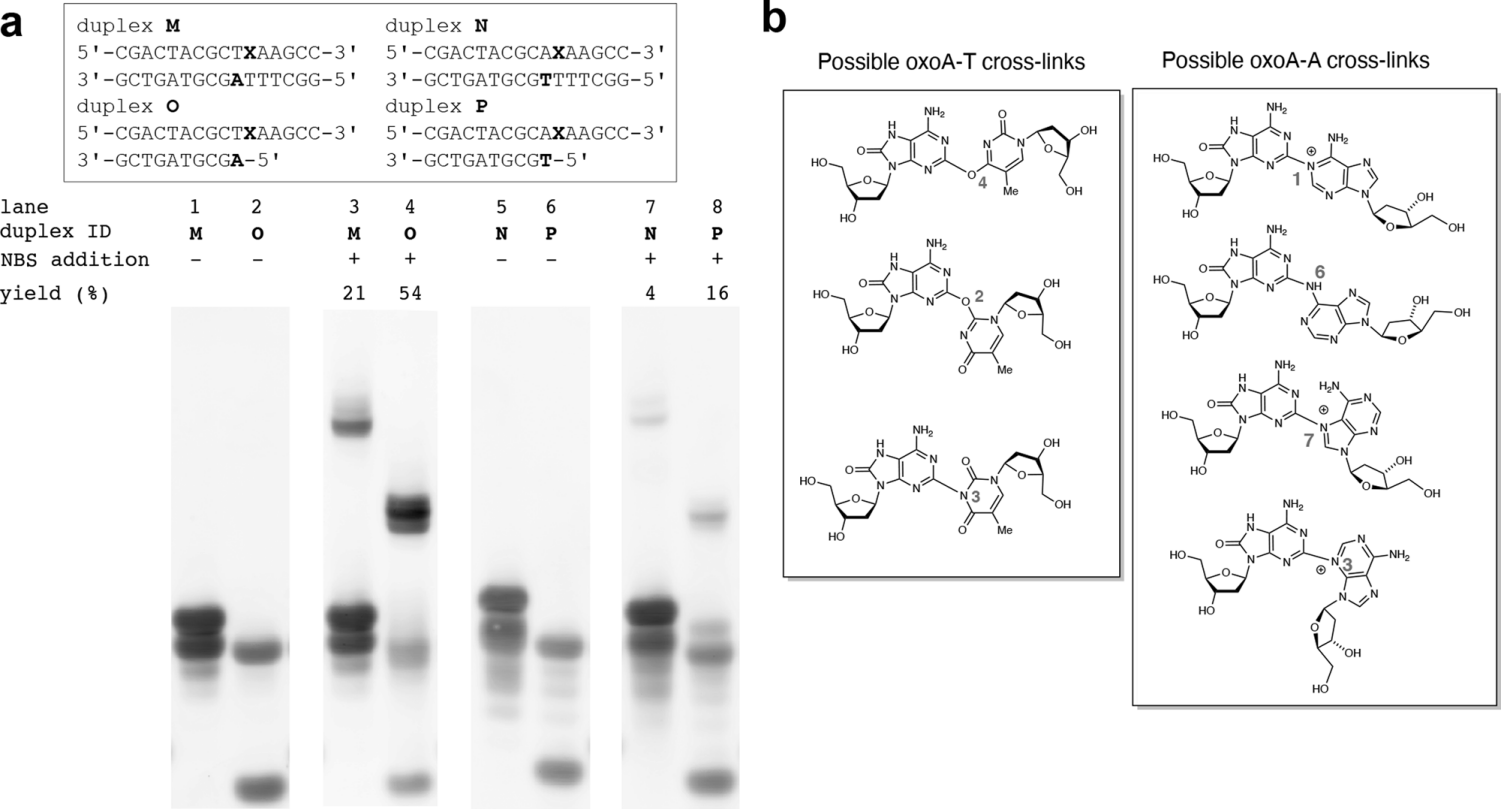
Denaturing PAGE analysis of NBS-catalyzed ICL reactions with modified duplexes M–P to identify secondary products formed alongside oxoA-A and oxoA-T ICLs. **a** PAGE analysis of cross-linking of DNA **M**–**P**. Duplexes contain 5′-ToxoA/AT-5′ (duplex **M**), 5′-AoxoA/TT-5′ (**N**) and recessed DNA containing 5′-ToxoA/A-5′ (**O**), 5′-AoxoA/T-5′ (**P**) sequences are shown. The yields of major oxoA ICLs from individual reactions are shown. Cross-linking yields were determined by dividing the percent band intensity of the cross-linked DNA band by the sum of the intensities of the unreacted complementary and oxoA strands. **b** Possible structures of oxoA-T and oxoA-A cross-links. Duplicate reactions were performed to ensure accurate reporting of yields and associated values. OxoA represents 8-oxoadenine and is defined as X in the figure. NBS *N*-bromosuccinimide, ICL interstrand cross-linking. The unmodified gel image for this figure is found in Supplementary Fig. 9.

To determine the identity of the minor bands present in both cross-linked duplex **B** and duplex **D**, we carried out a series of cross-linking reactions with duplexes **M**–**P** (Fig. 8). Duplexes **M** and **N** are the reverse sequences of duplexes **B** and **D**, respectively. Duplexes **O** and **P** consist of recessed DNA that have similar sequences as duplexes **M** and **N**, respectively, but lack the thymine residues paired with oxoA as well as all bases 3’ of the oxoA modifications (see Supplementary Fig. 9 for uncropped gel images). Removal of the thymine residues flanking the oxoA modification allows us to determine if the minor bands under investigation arise from a reaction with a nearby thymine residue or another functional group on the adenine (duplex **B**) or thymine moiety (duplex **D**), respectively. The reverse sequences of both duplexes were used because the complementary strands in duplex **O** and **P** needed to be long enough to prevent melting during the reactions.

NBS oxidation of duplex **M** resulted in a similar band migration pattern to what is observed upon oxidation of duplex **B** (Fig. 4) with minor bands forming along with the major band albeit at a slightly lower yield (21%, Fig. 8, lane 3). Treatment of recessed DNA **O** with NBS resulted in at least two major bands corresponding to cross-linked DNA that migrated at a very similar *R*_*f*_ (Fig. 8, lane 4). This suggests that another functional group on the adenine nucleobase participates in ICL formation along with the endocyclic N3. Interestingly, the combined yields of these bands were higher (54%) than that observed in the oxidation of duplex **B** (28%; Fig. 4, lane 4). The higher ICL yield and the formation of major bands could be attributed to the flexibility of the nucleophilic adenine at the 5’ end.

Oxidation of duplex **N** similarly resulted in two bands in the area expected of cross-linked DNA at a lower yield (4%, Fig. 8, lane 7) than that observed when treating duplex **D** with the same oxidant (8.9%, Supplementary Fig. 7). The same reaction carried out with recessed DNA **P** resulted in the formation of two bands at an elevated yield of 16% (Fig. 8, lane 8), which would be attributed to the flexibility of the nucleophilic thymidine residue at the 5’ end. These results suggest that the secondary band formed during the oxidation of duplex **D** arises from another functional group on the thymine ring. This conclusion is further supported by the PAGE analysis of cross-linked duplex **D** treated with hot piperidine. Treatment of the products from oxidation of duplex **D** with hot piperidine (200 mM, 90 °C) resulted in degradation of only the faster migrating cross-linked band (Supplementary Fig. 10, lane 10). The fact that only one band is piperidine labile indicates that the cross-linked products are unique. Overall, these results support the claim that both bands are oxoA-T cross-links arising from the involvement of different functional groups on the same thymine nucleobase.

### Sequence specificity for oxoA-mediated cross-linking

Our modeling of the sequences used in these cross-linking studies predicted that introducing the nucleophilic dG and dA residues of the opposite strand offset one base on the 5′-side of oxoA would place the putative reactive groups in the closest proximity (3.2 Å between C2 of oxoA and N2 of the 3′-dG [5′-CX/GT-5′, X = oxoA]; 3.2 Å between C2 of oxoA and N3 of the 3′-dA [5′-TX/AT-5′, X = oxoA], Fig. 2b), and thus promote cross-link formation. To evaluate how changing the position of the reactive nucleobases would affect ICL formation, we constructed duplexes **K** and **L**, which place the reactive base offset to the 3’-side of the oxoA modification and have the general sequence 5′-XM/TN-5′, where X is the electrophilic oxoA and N is the nucleophilic base. The PAGE analysis of NBS-treated duplexes **A**, **B**, **K**, and **L** is shown in Supplementary Fig. 11. Duplex **K** (5′-oxoAC/TG-5′) appears to have formed cross-links at a yield of 27.7 ± 0.4% (Supplementary Fig. 11, lane 6), which is ~10% lower than that of the analogous duplex **A** ICL yield (lane 5). The NBS oxidation of duplex **L** (5′-oxoAT/TA-5′) resulted in an ICL yield of 9.9 ± 0.5% (lane 8), which is lower than that of the duplex **B** reaction (28.4 ± 0.5%; lane 7). The decreased ICL yields can be explained by the helical structure of DNA placing the putative reactive groups further from the C2 position of oxoA in the 5′-XM/TN-5′ sequence context: 4.9 Å for duplex **K** (between C2-oxoA and N2-5′-dG) and 5.9 Å for duplex **L** (C2-oxoA and N3-5′-dA). Overall, these results indicate the oxoA cross-linking reaction displays a sequence preference in the general form of 5′-MX/NT-5′.

### Kinetic analysis of the oxoA cross-linking reactions

To further characterize the oxoA-mediated ICL formation, we determined kinetic parameters for the oxoA-G and oxoA-A cross-linking reactions in the presence of NBS or Na_2_IrCl_6_ (Supplementary Fig. 12). The oxoA-mediated cross-linking occurred rapidly with bands representing ICLs observed within 30 s of oxidant addition (gel not shown). The cross-linking reaction with dG occurred faster than the corresponding reaction with dA (2.5-fold for NBS-catalyzed reactions; 1.5-fold for the Na_2_IrCl_6_ reactions). The Na_2_IrCl_6_-catalyzed reactions occurred faster than those induced by NBS oxidation (twice as fast for reactions with dG; 3.5-times as quickly for reactions with dA). These results suggest the purine radical formed transiently during Na_2_IrCl_6_ oxidation is more reactive than the halogenated purine intermediate formed during RXS oxidation of oxoA.

### Thermal stability analyses of the oxoA cross-linked duplexes

To assess the impact of the cross-links on thermal stability of the oxoA-containing DNA duplexes, we determined melting temperatures for the oxoA-G cross-linked duplex **A** and the oxoA-A cross-linked duplex **B** (Supplementary Fig. 13). Introduction of the cross-link to duplex **A** increased the melting temperature by 22 °C relative to the non-cross-linked duplex (62.6 ± 0.2 °C vs. 84.6 ± 0.4 °C), while the oxoA-A ICL present in duplex **B** increased the melting temperature of by 20 °C (59.3 ± 0.2 °C vs. 79.3 ± 0.2 °C). The observed increase in melting temperature is consistent with the formation of ICLs and a greater thermal stability.

### Characterization of the oxoA cross-links by LC-MS analysis

We performed mass spectrometric analyses to further characterize the chemical structure of the oxoA cross-links in duplex DNA. Briefly, cross-linked duplex DNAs were digested with an enzyme cocktail containing benzonase, phosphodiesterase I, and alkaline phosphatase to release a fully digested cross-linked dinucleoside along with constituent nucleosides^44^. These mixtures were then enriched through HPLC and subjected to LC-MS analysis to identify the residues present in the cross-link.

**Fig. 9:**
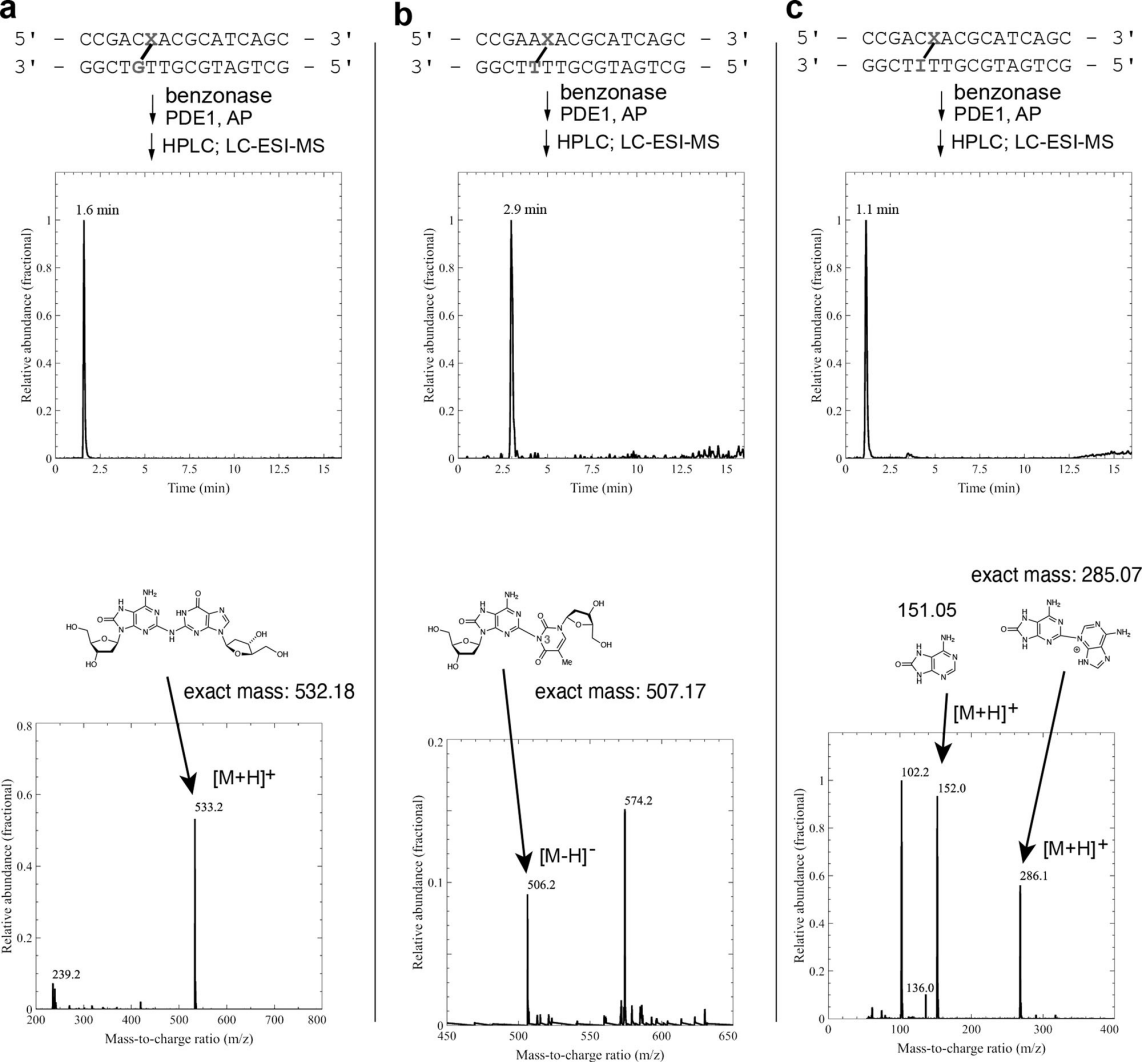
LC-ESI-MS analysis of the enzymatic digestion of duplexes A, D, and F. **a** Selected-ion chromatogram (SIC) and mass signal (MS) for digestion products of cross-linked duplex **A** (5’-CoxoA/GT-5’) with signal at m/z = 533.2 corresponding to a [M+H]^+^ ion of the fully digested oxoA-G cross-link remnant. **b** SIC and MS for digestion products of cross-linked duplex **D** (5’-AoxoA/TT-5’) with signal at m/z = 506.2 corresponding to a [M–H]^−^ ion and m/z = 574.2 corresponding to a [M-2H+Na+FA]^−^ ion of the fully digested oxoA-T cross-link remnant. A possible structure of the oxoA-T cross-links is shown. **c** SIC and MS for digestion products of cross-linked duplex **F** (5’-CoxoA/IT-5’) with signal at m/z = 286.1 corresponding to a [M+H]^+^ ion of cross-linked oxoA-I purine bases. A possible structure of the oxoA-I cross-links is shown. OxoA represents 8-oxoadenine and is defined as X in the figure. PDE1 phosphodiesterase I, AP alkaline phosphatase.

An LC-MS analysis of digested cross-linked duplex **A** revealed a product consistent with an oxoA-G cross-linked dinucleoside as shown in Fig. 9a (m/z 533.2, [M+H]^+^, Supplementary Fig. 14). This indicates that the cross-link that forms in duplex **A** indeed involves oxoA and guanine residues. Similar analysis of cross-linked duplex **B** did not reveal any peak corresponding to cross-linked dinucleosides or an explicit remnant thereof (e.g., cross-linked purine bases). However, LC-MS analysis of the HPLC peak, which is believed to be a fully digested, cross-linked oxoA-A dinucleoside, revealed signals that could correspond to the individual nucleosides oxoA and dA (Supplementary Figs. 15 and 16, m/z 288.1 [M^oxo-dA^+Na-2H]^−^, *m/z* 296.1 [M^dA^+HCOOH-H]^−^). This suggests that the oxoA-A cross-link is present but fragments upon ionization, which is consistent with the chemical instability of the major oxoA-A ICL product (Fig. 6, lane 10). Mass spectrometric analysis of the digested cross-linked duplex **C** did not reveal any meaningful signals corresponding to an oxoA-C cross-linked dinucleoside. This may be due to the relatively low yield of the duplex **C** cross-linking reaction and/or lower sensitivity of the mass spectrometer used for these analyses. LC-MS analysis of digested cross-linked duplex **D** showed two signals corresponding to an oxoA-T cross-linked dinucleoside (Fig. 9b and Supplementary Figs. 17 and 18 (m/z 506.2 [M–H]^−^). These results indicate that the cross-link formed in duplex **D** involves oxoA and thymine residues. Finally, the subjection of cross-linked duplex **F** to the same analyses revealed a signal corresponding to the remnants of a cross-linked oxoA-I dinucleoside in the form of covalently linked purine bases (Fig. 9c and Supplementary Fig. 19 (m/z 286.1 [M+H]^+^). Furthermore, the individual purines (i.e., oxo-adenine and HX) were also detected (Fig. 9c and Supplementary Fig. 19, m/z 152.0 [M^oxoA^+H]^+^ and m/z 136.0 [M^HX^+H]^+^), which indicates that the cross-link formed in duplex **F** involves oxoA and inosine residues.

## Discussion

### OxoA forms cross-links under physiologically relevant conditions

Here, we report that the major oxidative adenine lesion, oxoA, can produce ICLs with both dG and dA residues in moderate to high yields (up to 62%) in duplex DNA upon treatment with physiologically relevant oxidants in vitro. DNA ICLs represent one of the most genotoxic lesions encountered in nature; as few as 20 unrepaired ICLs have been shown to kill mammalian cells^45^. This form of damage is particularly relevant to the pathology of the rare genetic disorder, FA, as patients suffering from this disorder are unable to repair ICL damage. Therefore, identifying forms of these ICLs that form via exogenous or endogenous sources is warranted. Reported endogenous DNA ICLs include those formed by abasic sites, oxidized abasic sites, unsaturated aldehydes, nitric oxide, and the bacterial toxin, colibactin^23,25,32,33,46,47^. The ICLs reported in this work involve one of the most common forms of base damage, oxoA, and the yield of the oxoA-G ICL is higher than other reported endogenous cross-links, with the exception of the abasic site-A ICL (~70%)^24^. These oxoA ICLs appear to form at faster rates (detectable amounts of ICL observed after 30 s) than the abasic site-A ICL (detectable amounts observed at 1 h)^24^. These oxoA ICLs can form through two separate, physiologically relevant oxidation mechanisms: RXS oxidation (associated with the inflammatory response) and one-electron oxidation, which highlights the lesion’s cross-linking reactivity. Furthermore, the lower redox potential of oxoA (0.92 V) compared to the canonical nucleobases (e.g., 1.29 V (G), 1.42 V (A)) makes it more likely that a second oxidation event will occur, which increases the likelihood of the formation of these ICLs^5^. Taken together, oxoA appears capable of forming ICLs with various nucleobases under a variety of physiologically relevant conditions in vitro, which suggests that these cross-links may form in the cellular environment.

### OxoA cross-links with guanine residues under in vitro conditions that mimic the inflammatory response

Perhaps the most interesting result from our oxoA cross-linking screens is the detection of the oxoA-G ICLs induced by HOCl oxidation through both NaOCl in solution and the MPO/H_2_O_2_/Cl system, which mimic the oxidative conditions of chronic inflammation (Fig. 4, lane 6). An important step in the human inflammatory response involves the neutrophil-mediated release of enzymes capable of generating RXS to kill pathogens. The formation of RXS is carried out by MPO, which utilizes H_2_O_2_ and chloride anions to generate HOCl. Acute inflammatory responses generally do not result in negative outcomes to the cell, but prolonged responses associated with chronic inflammation have been shown to result in significant intracellular oxidation damage to proteins, lipids, and DNA, often resulting in apoptosis, necrosis, and tumorigenesis^3,37,48^. In fact, this prolonged inflammation is considered to be a cause for increased incidences of cancer initiation in persons suffering from chronic inflammatory diseases^48^.

The extracellular concentration of MPO released by neutrophils during the inflammatory response is in the low-millimolar range, while H_2_O_2_ extracellular concentrations may be up to 100 micromolar during oxidative distress^36,49^. The extracellular concentrations of hypochlorite are estimated to be on the order of high-micromolar to low-millimolar^36,50^. Estimates of the intracellular concentration of HOCl during the inflammatory response have been difficult to determine, as hypochlorite has been shown to quickly react with protein thiols and methionine residues on the cell membrane surface^36^. However, HOCl transport into the intracellular environment has been well documented as it has been shown to react with reduced glutathione and generate halogenated DNA adducts in the form of 8-chloroguanine (8-Cl-G), 8-chloroadenine (8-Cl-A), and 5-chlorocytosine (5-Cl-C) in a cellular environment^51–55^. In fact, treating various cell lines with physiological concentrations (extracellular) of HOCl observed during the inflammatory response has been shown to form up to six 5-Cl-C lesions per 10^6^ dC, which demonstrates the extent of potential DNA damage caused by the oxidant^55^. While chloropurine damage was not directly observed under these conditions, elevated amounts of oxoG and oxoA were found, which suggests that 8-Cl-G and 8-Cl-A were generated as transient lesions^55,56^. While the concentrations of hypochlorite utilized in this report (up to 1 mM) are almost certainly an exaggeration relative to the observed intracellular concentrations, the facile formation of the oxoA ICLs in the presence of the MPO/H_2_O_2_/NaCl system suggests that oxoA cross-linking may occur endogenously under conditions of chronic inflammation given the reactivity documented herein. This finding is notable, as it attaches greater biological significance to the oxoA-mediated ICLs, and given the mutagenicity and cytotoxicity of ICLs may help further our understandings of apoptotic, necrotic, and carcinogenesis mechanisms associated with chronic inflammation.

### Implications of the structural and stability variations among the oxoA cross-linking lesions

OxoA is capable of cross-linking with multiple nucleobases (e.g., G, A, C, T, I; Fig. 4, lanes 3 and 7, Fig. 5, lanes 5 and 11, Supplementary Fig. 7, lanes 7–12) to produce structurally diverse ICLs. ICLs typically vary in the groove-position of the covalent linkage, helical distortion of the DNA duplex, and chemical stability. The results of our base substitution reactions (Fig. 5) suggest that the oxoA cross-linking reactions with G and A residues mainly occur in the minor groove of DNA, where the C2 of oxoA is linked to the N2 of guanine and the N3 of adenine, respectively (Fig. 7).

Thermal stability analyses of oxoA-G and oxoA-A ICL DNA show that the two cross-links variably stabilize the duplexes, with the oxoA-G ICL displaying higher stability of the duplex than the oxoA-A cross-link (Δ*T*_*m*_ = 22.0 vs. 20.0 °C, respectively; Supplementary Fig. 13). This is likely due to the distortion of the helical structure by covalent linkages at variable degrees, thus interrupting hydrogen bond interactions near the cross-link in different ways. Furthermore, this helps explain the observed difference in migration patterns between the oxoA-G and oxoA-A ICLs, as this difference in helical distortion likely results in altered three-dimensional structures.

**Fig. 10:**
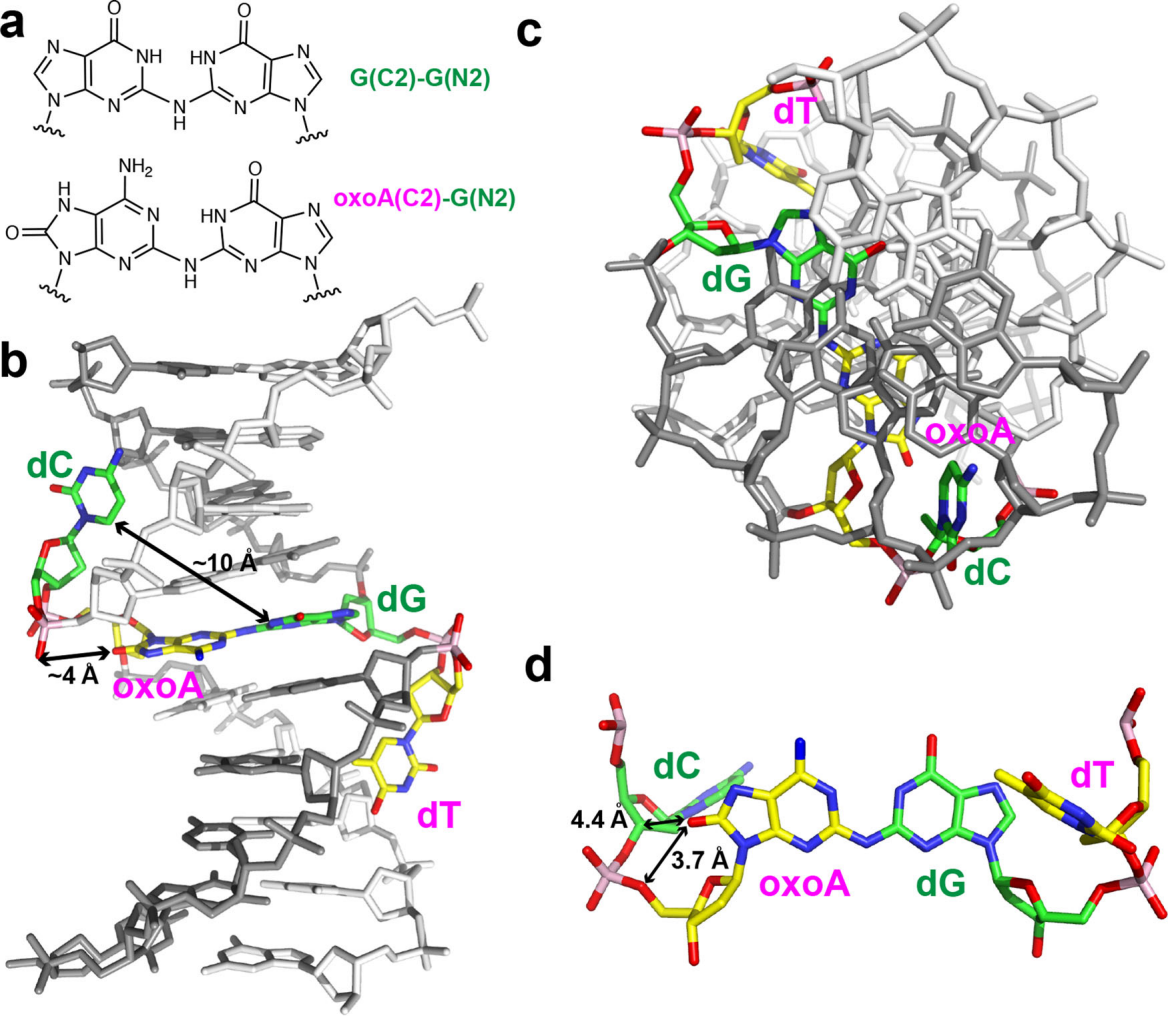
Modeled structure of duplex DNA containing an oxoA-G interstrand cross-link. The model was generated by modification of a solution structure containing a G(C2)-G(N2) cross-link in the 5’-CG/GC-5’ sequence context (PDB code: 1S9O [10.2210/pdb1S9O/pdb]). **a** Comparison of the oxoA(C2)-G(C2) cross-link with G(C2)-G(N2) cross-link. **b** Side view of the oxoA-G cross-link-containing DNA. dG and dC that can base pair in non-cross-linked DNA are in green/natural atom. OxoA and dT that can base pair in non-cross-linked DNA are in yellow/natural atom. Note that the dC and dT are flipped out and reside in the minor groove that is widened. The oxoA-G cross-link would adopt a near coplanar geometry and stack well with spatially adjacent base pairs. **c** Top view of oxoA-G cross-link-containing DNA. **d** Top view of cross-linked 5’-CoxoA/GT-5’ only. The distance between the dC(C5) and dG(N1) is indicated. Distances between 8-oxo moiety of oxoA and nearby atoms is indicated. Note that N1 of oxoA and N1-H of G can form a hydrogen bond, which can further stabilize the cross-link. OxoA 8-oxoadenine.

The published solution structure of G(C2)-G(N2) cross-link-containing duplex DNA (PDB code: 1S9O)^57^ provides insights into the three-dimensional structure of our oxoA-G ICL. Nitrous acid generates guanine-guanine ICLs in the 5’-CG/GC-5’ sequence context, where an NH moiety is covalently linked to the C2 atoms of the two guanines (Fig. 10a). The chemical structure of the G(C2)-G(N2) cross-link in duplex DNA would be very similar to that of the oxoA-G cross-link (Fig. 10a). In both structures, the C2 atoms of two purines are connected by an NH moiety. These cross-links form in the minor groove of B-form DNA with a similar linkage as the oxoA-G ICL reported here^57^. In addition, in both the 5’-C(oxoA)/GT-5’ and the 5’-CG/GC-5’ ICLs, the two 3’ purines engage in the cross-linking reaction. Furthermore, structural analysis suggests that the distance between N1s of the two guanines in the G(C2)-G(N2) ICL would be similar to that between N1s of the two purines in the oxoA-G ICL: The NMR structure of the G(C2)-G(N2) ICL shows a distance of 2.7 Å for the two guanine N2s, which cannot occur if both guanines are in their major keto tautomers due to repulsive steric interaction. In turn, this suggests that one of the two guanines exists as the minor enol tautomer, which would allow the formation of the observed hydrogen bond between N1s of the guanines. In the case of the oxoA-G ICL, the N1s of oxoA and G can form a hydrogen bond in their major tautomeric conformations (Fig. 10a). The comparison between the oxoA-G and G-G cross-links leads us to propose that the oxoA-G ICL structure would be very similar to the G(C2)-G(N2) ICL structure. Therefore, we built a modeled structure of the oxoA-G ICL with modification of the known G(C2)-G(N2) ICL structure (Fig. 10b–d). As similarly observed in the G(C2)-G(N2) ICL structure, the oxoA-G ICL structure would display a near coplanar oxoA-G cross-link; a widened minor groove near the cross-link; B-DNA-like conformation; favorable stacking interactions among the oxoA-G cross-link and spatially adjacent base pairs; base flipping of the pyrimidines opposite oxoA and the G; and stabilization of the flipped-out pyrimidines via interaction with the widened minor groove.

The stabilization of extrahelical bases would not be observed should the cross-link forms between bases in opposing grooves^57,58^. While the above thermal stability analyses were not carried out on the oxoA-C or oxoA-T ICLs, we would expect these cross-links to result in different helix stabilization if the linkages occur in different grooves of the DNA helix^59–62^. Lastly, the chemically unstable N3 linkage present in the oxoA-A ICL would make it susceptible to hydrolytic cleavage of the glycosidic bond and formation of secondary lesions, such as abasic sites and single-strand DNA breaks^63,64^.

If the oxoA ICLs are indeed produced endogenously, the structurally diverse oxoA ICLs would be recognized and resolved by a variety of cellular repair mechanisms with different efficiencies. ICLs that stall the replisome are most commonly repaired by the FA pathway, which involves three crucial steps: un-hooking of the cross-link by incision of one parental strand, translesion synthesis (TLS) past the un-hooked mono-adduct, and finally gap-filling through homologous recombination^20^. The groove-position of the covalent linkage plays a significant role in how the TLS bypass step is carried out^65,66^. TLS is notably an erroneous, mutagenic process. Given the fact that oxoA can form cross-links at observable yields in both the major and minor grooves, we speculate that various TLS polymerases might be involved in the repair of the oxoA ICLs, thereby inducing variable mutagenic stress upon the cell. As helical distortion affects the efficiencies of both the un-hooking and TLS steps in FA repair, the oxoA-G and oxoA-A ICLs would be repaired at different efficiencies^65,67^. The ability of oxoA to form structurally diverse cross-links suggests that various ICL repair mechanisms within the cell may be induced, depending on the identity of the cross-link, to excise the various oxoA ICLs in conditions of chronic inflammation or oxidative stress. Detailed understandings of the cellular responses to these particular ICLs require further investigations.

### The mutagenic properties of the major oxidative adenine lesion

Our studies of the oxoA ICLs provide insights into the mutagenicity of oxoA. While oxoA has been known to be one of the two major oxidative lesions, the mutagenic properties of oxoA have not been as thoroughly studied as those of oxoG. This knowledge gap is in part due to early reports of the accurate bypass of oxoA by prokaryotic DNA polymerases, as well as reports of a low demonstrated mutagenicity of oxoA in prokaryotes^68^. Later studies showed that oxoA is significantly mutagenic in mammalian cells, underscoring the differential mutagenicity of oxoA in prokaryotes and eukaryotes. Indeed, recent kinetic and structural studies reveal that bypass of oxoA by human DNA polymerases is highly error prone^69,70^. Unlike oxoG, the mutagenicity of oxoA in human cells may be partially due to minor groove contacts by DNA polymerases^69,70^. The cross-linking reactivity of oxoA reported here highlights chemistry specific to oxoA, in comparison to oxoG. This difference is likely a consequence of the intrinsic reactivity of oxoA compared to oxoG, as oxoG has been shown to readily form secondary lesions upon oxidative stress in the form of spiroiminodyhydantoin and guanidinohydantoin^38,71^. These lesions have not been observed when oxoA was subjected to similar oxidative conditions, suggesting the formation of adducts at the C2 position may be the preferred pathway^13^. The high cellular levels of oxoA, as well as the lesion’s ability to form ICLs, implicate the lesion as a considerable threat to cellular health. Interestingly, the ability of oxoA to form ICLs may be linked to the pathology of the rare genetic disorder, FA. It has been observed that FA cells, which are deficient in ICL repair, are sensitive to oxidative stress, which might be partially explained by oxoA-induced cross-linking^28–31^. The results from our studies herein warrant more attention to the genotoxic characteristics of the oxoA lesion.

### OxoA may cross-link with varying moieties under identical sequence contexts

PAGE analysis of the cross-linking reactions involving oxidation of duplexes **B** and **D** reveals multiple bands corresponding to DNA cross-links (Fig. 4 and Supplementary Fig. 7, lanes 10–12). Preliminary analyses comparing the yields and band migration patterns among all duplexes studied suggest that the major bands formed in the reactions of duplexes **B** and **D** correspond to oxoA-A and oxoA-T cross-links, respectively. However, identifying the source of the minor bands that migrated above the major products requires interpretation of multiple data points presented in this report. For both duplexes, it seems most likely that these secondary bands arise from either a cross-linking between oxoA and a nearby base that is neither the adenine (5’-ToxoA/AT-5’, duplex **B**) nor thymine (5’-AoxoA/TT-5’, duplex **D**), or a cross-linking between oxoA and other functional groups of the adenine (5’-ToxoA/AT-5’, duplex **B**) or thymine (5’-AoxoA/TT-5’, duplex **D**) moieties.

To elucidate the identity of the minor bands arising from duplex **B** oxidation, we can first look at the reactions involving modified base-containing duplexes **I** and **J**, which have **7**-deazaadenine and 3-deazaadenine in place of adenine, respectively (Fig. 5). The reaction between oxoA and 3-deazaadenine in duplex **J** results in almost a complete loss of the major band (Fig. 5, lane 14) observed when duplex **B** is treated with NBS (Fig. 5, lane 11), which suggests that the major band arises from oxoA cross-linking with the endocyclic N3 of adenine. However, we still observe the minor bands when duplex **J** is oxidized (Fig. 5, lane 14), which suggests that the minor bands arise from another functional group on the adenine moiety or from a nearby base. Next, we can observe that, when cross-linked duplex **B** is treated with piperidine, the cross-link giving rise to the minor band is not degraded while the major cross-linking product is lost (Fig. 5, lane 10). This provides further evidence that these bands are structurally unique. This conclusion is further supported by the oxidation products of recessed DNA **O** (Fig. 8). PAGE analysis of the oxidation products of recessed DNA **O**, which removed the nearby thymine residues from the reverse sequence of duplex **B** (duplex **M**), resulted in at least two bands corresponding to cross-linked DNA migrating at a similar *R*_*f*_ (Fig. 8, lane 4). Since the nearby dT nucleotides were removed from the reaction and two bands still formed, it follows that these bands would be oxoA-A cross-links with linkages at different positions on the purine ring of adenine. These positions could feasibly be the N1, N6, or N7 positions of adenine, but we have made no attempts in this report to elucidate this linkage. Overall, these observations strongly suggest that the minor cross-linked products of duplex **B** oxidation may arise from reactions occurring between the C2 of oxoA and other nucleophilic groups of the adenine. Nevertheless, these results do not rule out the possibility that nearby thymine residues compete in the formation of cross-links.

To identify minor bands formed during oxidation of duplex **D**, we can first observe that NBS treatment of recessed DNA **P** (Fig. 8), which removed adjacent thymine residues from the reverse sequence of duplex **D** (duplex **N**), results in two bands corresponding to cross-linked DNA (Fig. 8, lane 8). This suggests that the source of the secondary bands formed upon duplex **D** oxidation is another functional group on the same thymine ring. This conclusion is further supported by the observation that treatment of cross-linked duplex **D** with hot piperidine results in loss of only one cross-link band upon PAGE analysis (Supplementary Fig. 10), which indicates that the cross-links are structurally unique from one another. Thymine adducts are not prevalent in the literature when compared to the purines, but precedent exists for the formation of O2, O4, and, to a lesser extent, N3 covalent linkages^72–74^. We speculate that the O2, O4, and N3 positions are likely to be responsible for the oxoA-T cross-linking products observed upon oxidation of duplex **D**^75^. Furthermore, the cross-links involving the O2-, O4-, and N3-thymine adducts would exhibit different chemical stabilities, which may help explain the persistence of one cross-linked band upon piperidine treatment of cross-linked duplex **D** (Supplementary Fig. 10)^76^. It must be acknowledged that only one peak was observed in the area we would expect a cross-linked dinucleoside to elute during the HPLC purification of an enzymatically digested crude, duplex **D** cross-linking reaction (Supplementary Fig. 17). It is possible that the minor cross-linking product of duplex **D** oxidation forms at too low a yield to be identified by HPLC, or it may be a consequence of the two cross-links differing chemical stabilities. Given these observations, we conclude that the cross-linking products arising from the oxidation of duplex **D** may be two oxoA-T cross-links that are linked at different positions on the same pyrimidine ring of thymine.

We have discovered that under oxidative conditions, oxoA, but not 8-oxoG, readily reacts with dA and dG to generate cross-links, highlighting the specificity of 8-oxopurine-induced ICL formation. OxoA acts as a latent alkylating agent that undergoes an oxidative activation and reacts with an opposite nucleobase to produce ICLs. The oxoA ICLs represent an example of ICLs involving oxidized nucleobases. The oxoA-mediated cross-linking reaction can occur in the presence of various oxidants, including RXS and one-electron oxidants. The oxoA-G ICL is also produced in the MPO/H_2_O_2_/Cl^−^ system that mimics inflammatory conditions, suggesting the oxoA ICLs may form endogenously, especially in chronic inflammation. A probable mechanism involves a further oxidation of oxoA into a reactive iminoquinone, followed by the nucleophilic attack of an opposite base (dA, dG, dT, or dC) onto the C2 of the iminoquinone. Our mechanistic studies suggest the N2 of guanine and N3 of adenine attack the C2 of oxoA to produce the major oxoA-G and oxoA-A ICLs, respectively. LC-MS analyses confirmed the participating nucleobases in the oxoA-G, oxoA-T, and oxoA-I cross-links, respectively. Preliminary analyses of the secondary products formed alongside the oxoA-A and oxoA-T ICLs, respectively, suggest that other functional groups on the adenine and thymine moieties may be capable of cross-linking in the context of oxoA-A and oxoA-T ICLs. The oxoA-mediated ICL reaction preferentially occurs in the 5′-M(oxoA)/NT-5′ sequence, where M/N are C/G or T/A. Together, the facile formation of the oxoA ICLs along with the reported prevalence of oxoA in cells experiencing oxidative stress suggests that oxoA-mediated ICLs may occur in vivo. Our findings provide insights into oxoA-mediated mutagenesis. These results also further our understandings of endogenous ICLs, as well as the possible relationship between oxidative stress and the oxidation-induced formation of endogenous ICLs. Furthermore, these findings may be used in developing latent cross-linking drugs that selectively target the tumor microenvironment, which commonly exhibits elevated levels of ROS.

## Methods

8-Oxoadenine (oxoA), 8-oxoguanine (oxoG), 7-deazaguanine (7ZG), 7-deazaadenine (7ZA), and 3-deazaadenine (3ZA) phosphoramidites for modified ODN synthesis were purchased from Glen Research (Sterling, VA, USA). Single-stranded ODNs containing oxoA, oxoG, 7ZG, 7ZA, and 3ZA modifications were custom-synthesized by Midland Certified Reagent Co. (Midland, TX, USA). Unmodified and inosine-modified ODNs were purchased from Integrated DNA Technologies (Coralville, IA, USA). All ODNs used in these experiments were purified by PAGE prior to use. C-18 Sep-Pak cartridges were purchased from Waters (Milford, MA, USA). C-4 ZipTip pipette tips were purchased from MilliporeSigma (Burlington, MA, USA). Human MPO was purchased from R&D Systems (Minneapolis, MN, USA). Quantification of all DNA samples was performed on a NanoDrop 2000c spectrophotometer (Thermo Fisher Scientific, Waltham, MA, USA). Imaging of polyacrylamide gels was performed on a Typhoon FLA 9000 Gel Imager (GE Healthcare Life Sciences, Boston, MA, USA). Gel image quantification was done with ImageQuant software (GE Healthcare Life Sciences, Boston, MA, USA). MALDI-TOF-MS analyses were performed on a Bruker MALDI-TOF/TOF autoflex maX (Bruker, Billerica, MA, USA). Thermal stability experiments were performed on an Evolution 260 Bio UV-Vis spectrophotometer (Fisher Scientific, Waltham, MA, USA). Molecular modeling for design of experimental DNA duplexes was constructed using UCSF Chimera and based on PDB entry 1BNA. All other materials and reagents used were purchased from Fisher Scientific. Unless otherwise noted, all data are reported as mean ± SEM of three replicates.

### Procedures for oxoA cross-linking reactions with NBS, NaOCl, and Na_2_IrCl_6_ in duplex DNA

dsDNA duplexes were prepared by annealing the PAGE-purified oxoA-modified ODNs with 1 molar equivalent of their respective complementary ODNs (50 μM) in 10 mM sodium phosphate buffer (pH 7) and 100 mM NaCl at 90 °C for 5 min with slow cooling to room temperature overnight. These duplexes and their constituent ODNs can be found in Fig. 2b. All reactions were carried out at 37 °C and performed in triplicate. Stock solutions of NBS (0.4 mM, 1 molar equiv./μL) were prepared from pure, recrystallized NBS prior to every set of reactions. Stock solutions of NaOCl (4 mM, 10 molar equiv./μL) and sodium hexachloroiridate (IV) (Na_2_IrCl_6_) (16 mM, 200 molar equiv./5 μL) were prepared fresh from supplier stocks prior to every set of reactions. Reaction mixtures were prepared by diluting the stock dsDNA to a final concentration of 26.7 μM (15 μL, 0.4 nmol) with annealing buffer (10 mM phosphate buffer, pH 7, 100 mM NaCl) and incubating at 37 °C for 5 min. Reactions were initiated by adding oxidant. For NBS- and NaOCl-treated reactions, 1 μL of oxidant stock was added every 15 min for 1.25 h (5 total equivalents for NBS, 50 total equivalents for NaOCl). For Na_2_IrCl_6_-treated reactions, 5 μL of stock was added at *t* = 0 (200 total equivalents) and the reaction was allowed to proceed for 1.25 h. Reactions were stopped by adding 1 volume of formamide loading buffer (95% formamide, 5 mM EDTA, 0.025% (w/v) bromophenol blue), and samples were loaded onto a 20% denaturing polyacrylamide gel. The gel was electrophoresed at 250 V for 3 h, then stained with SYBR Gold Nucleic Acid gel stain for 1 h with gentle rocking. Stained gels were then imaged and quantified to determine cross-linking yields. Cross-linking yields were determined by two methods: (1) dividing the percent band intensity of the cross-linked DNA band by the sum of the intensities of the unreacted ODN bands based on ImageQuant analysis; (2) quantification of PAGE-purified, gel-extracted cross-link DNA on a NanoDrop. Gel image quantification was done with ImageQuant software (GE Healthcare Life Sciences, Boston, MA, USA). All yields and related statistics were derived from independent experiments and reported as the mean value ± SEM based on three independent replicates.

### Procedures for oxoA cross-linking reactions with human myeloperoxidase, H_2_O_2_, and NaCl

DNA duplexes were prepared as described above. All reactions were carried out at 37 °C and performed in triplicate. Stocks of human MPO were prepared by dissolving the lyophilized stocks in ultra-pure distilled water per the supplier’s recommendation. Reaction mixtures were prepared by adding H_2_O_2_ (final concentration of 250 μM), diluting with annealing buffer (final DNA concentration of 20 μM and NaCl concentration of 40 mM), and equilibrating at 37 °C for 3 min before adding MPO to a final concentration of 50 nM to initiate the reactions (total volume = 20 μL). Reactions were run for 1.25 h at 37 °C and quenched with 1 volume of formamide loading buffer. These reactions were then analyzed via denaturing urea PAGE as described above. Note that no gel extraction yields were calculated for this set of reactions. All yields and related statistics were derived from independent experiments and reported as the mean value ± SEM based on three independent replicates.

### General procedure for gel extraction and purification of DNA

Denaturing polyacrylamide gels (20%) were prepared and run as described above. For purifications of ODNs, the gels were run for 1.5 h. After PAGE separation, the bands of interest were excised from the gel using a razor blade, crushed using a small-pore syringe, resuspended in 1X TE buffer, subjected to a rapid freeze-thaw cycle (liquid nitrogen followed by rapid thaw for 5 min in a 90 °C water bath), and eluted overnight at room temperature on a rotary shaker. The eluent was then syringe filtered to remove gel fragments and concentrated on a vacufuge (Eppendorf, Hamburg, Germany). Samples for MALDI-TOF-MS analysis were ethanol precipitated, dried on a vacufuge, and stored at −20 °C. Samples used for all other analyses were then desalted on a C-18 Sep-Pak column following standard procedures. The purified DNA duplexes were quantified, dried on a vacufuge, and stored at −20 °C.

### Piperidine work-up of cross-linked duplexes

NBS-induced cross-linking reactions were performed as described above then filtered to remove excess oxidizing agent (3 kDa MWCO centrifugal filtration), dried on a vacufuge, and resuspended in 200 mM piperidine. Control reactions were prepared by resuspending the same filtered, dried reaction mixtures in ultra-pure distilled water. The piperidine-containing solution and control solution were then incubated at 90 °C for 30 min. Upon completion of the reaction, the samples were then cooled on ice for 5 min before removing the solvent on a vacufuge (Eppendorf). The evaporated samples were resuspended in formamide loading buffer and subjected to PAGE analysis as described above.

### Procedure for MALDI-TOF-MS analysis of cross-linked duplexes

Ethanol-precipitated DNA was resuspended in ultra-pure distilled water to a concentration of 20 μM and desalted via C-4 ZipTip pipette tips using ultra-pure distilled water for washing and 50% (v/v) ACN for elution. Once desalted, 1 μL (200 pmol) of sample was dispensed on the sample plate and allowed to evaporate completely at room temperature. Once dried, 1 μL of matrix solution (30 mg/mL 3-hydroxypicolinic acid, 10 mg/mL ammonium citrate in 1:1 acetonitrile:water solution) was spotted at the same location on the sample plate and allowed to evaporate completely. The sample was analyzed via matrix-assisted laser desorption/ionization time-of-flight mass spectrometry (MALDI-TOF-MS) in linear mode at 85% maximum voltage. ODNs of known molecular weight (6–12 kDa) were used as standards for calibration and prepared using identical methods. Data were analyzed using Bruker autoFlex maX analysis software. All MALDI-TOF-MS analyses were performed in duplicate to ensure accurate reporting of observed mass values.

### Thermal stability analysis of cross-linked and non-cross-linked DNA duplexes

DNA duplexes for thermal stability analyses were annealed at a concentration of 5 μM in 100 mM NaCl and 10 mM sodium phosphate buffer (pH 7). All DNA melting curves were monitored at 260 nm from 30 to 95 °C with a temperature gradient of 1.0 °C/min and performed in triplicate. Duplex melting temperatures were calculated from the first derivative of the melting curves by the Thermo INSIGHT analysis software. All melting temperatures and related statistics were derived from independent experiments and reported as the mean value ± SEM based on three independent replicates.

### Kinetic analysis of the oxoA cross-linking reactions

Duplex DNA and reaction mixtures were prepared as described above. All reactions were performed in triplicate. Reactions were initiated by adding 5 μL of oxidant (5 equiv. NBS or 100 equiv. Na_2_IrCl_6_; 20 μL total reaction volume). Reactions were quenched at the desired time points by adding 1 volume of formamide loading buffer and stored at −20 °C until denaturing urea PAGE analysis. The denaturing PAGE analysis, gel staining, and yield quantification were performed as described previously. ICL yields were plotted in Microsoft Excel and fit to a first-order rate equation through nonlinear regression ($Y = {\mathrm{A}}^\ast \left( {1 - \exp \left[ { - {\mathrm{b}}^\ast t} \right]} \right)$ (1), where *Y* is the percent ICL yield; A is defined as the maximum cross-linking yield; and b is defined as the rate constant, *k*_*ICL*_). The reaction half-life (*t*_1/2_) was determined using the following equation: $t_{1/2} = - \frac{1}{{k_{ICL}}}{\mathrm{ln}}\left( {\frac{1}{2}} \right)$ (2). All yields, related kinetic values, and statistics were derived from independent experiments and reported as the mean value ± SEM based on three independent replicates.

### Enzymatic digestion of cross-linked duplexes

Cross-linked duplexes **A**, **B**, **D**, and **F** were digested using an enzyme cocktail under conditions described by Quinlivan and Gregory with slight modifications^44^. Benzonase (5 units), alkaline phosphatase (5 units), phosphodiesterase I (0.005 units), and EHNA (5 nmol) were added to 10 nmol of cross-linked duplex in solution with 10 mM MgCl_2_, 50 mM NaCl, and 10 mM Tris-HCl, pH 7.9. The purpose of EHNA in this context was to act as an inhibitor for deamination of dA to dI induced by residual adenine deaminase present in commercial enzyme stocks. This mixture was incubated at 37 °C overnight. Upon completion, the digestion mixture was extracted with chloroform to remove enzymes, and the aqueous layer was concentrated on a vacufuge, resuspended in ultra-pure distilled water, and purified via HPLC.

### HPLC purification of enzymatic digestion mixtures with LC-MS analysis

Purifications of the enzymatic digestion products were carried out on a 4.6 mm × 250 mm Thermo Scientific C18 column (5 μm particle size) using 0.1 M TEAA (containing 5% v/v ACN) and ACN as mobile phases with the following linear gradient: 0–30% ACN over 30 min, 30–100% ACN over 3 min, and 100% ACN over 5 min. Fractions were collected every 30 s with fractions of interest being collected and concentrated on a vacufuge. These concentrated fractions were then injected onto a 50 mm × 2.1 mm ZORBAX Eclipse Plus C18 column (5 μm particle size) using 0.1% (v/v) formic acid in water (solution A) and 0.1% (v/v) formic acid in methanol (solution B) as mobile phases with the following linear gradient: 0–100% B over 16 min. The eluent from LC separation was analyzed by a single quadrupole mass spectrometer (Agilent Technologies) with an electrospray source. All mass spectrometry data were analyzed using Agilent ChemStation software. All enzymatic digestions and associated LC-MS analyses were performed in duplicate to ensure accurate reporting of data.

### Reporting summary

Further information on research design is available in the Nature Research Reporting Summary linked to this article.

## Supplementary information

Supplementary Information

Reporting Summary

## Data Availability

The authors declare that all data supporting the findings of this study are available within the paper and its Supplementary information files. Source data are provided with this paper.

## Source data

Source Data
