# Supplementary material for: DNA interstrand cross-links induced by the major oxidative adenine lesion 7,8-dihydro-8-oxoadenine
Source: Nat Commun. 2021 Mar 26;12:1897. doi: 10.1038/s41467-021-22273-2 (PMC7997976; doi:10.1038/s41467-021-22273-2)
Supplement: Supplementary file 1 — Supplementary Information [file 41467_2021_22273_MOESM1_ESM.pdf]

Supplementary Information

Endogenous DNA interstrand cross-links induced by the major oxidative adenine lesion 7,8-dihydro-8-oxoadenine

Aaron L. Rozelle[1,2], Young Cheun[1], Caroline K. Vilas[1], Myong-Chul Koag[1] and Seongmin Lee[1*]

[1] Division of Chemical Biology and Medicinal Chemistry, College of Pharmacy,
The University of Texas at Austin, Texas 78712, United States

[2] McKetta Department of Chemical Engineering, Cockrell School of Engineering, The University of Texas at Austin, Austin, Texas 78712, United States

*E-mail: seongminlee@austin.utexas.edu, Tel: 1-512-471-1785

Supplementary Figures

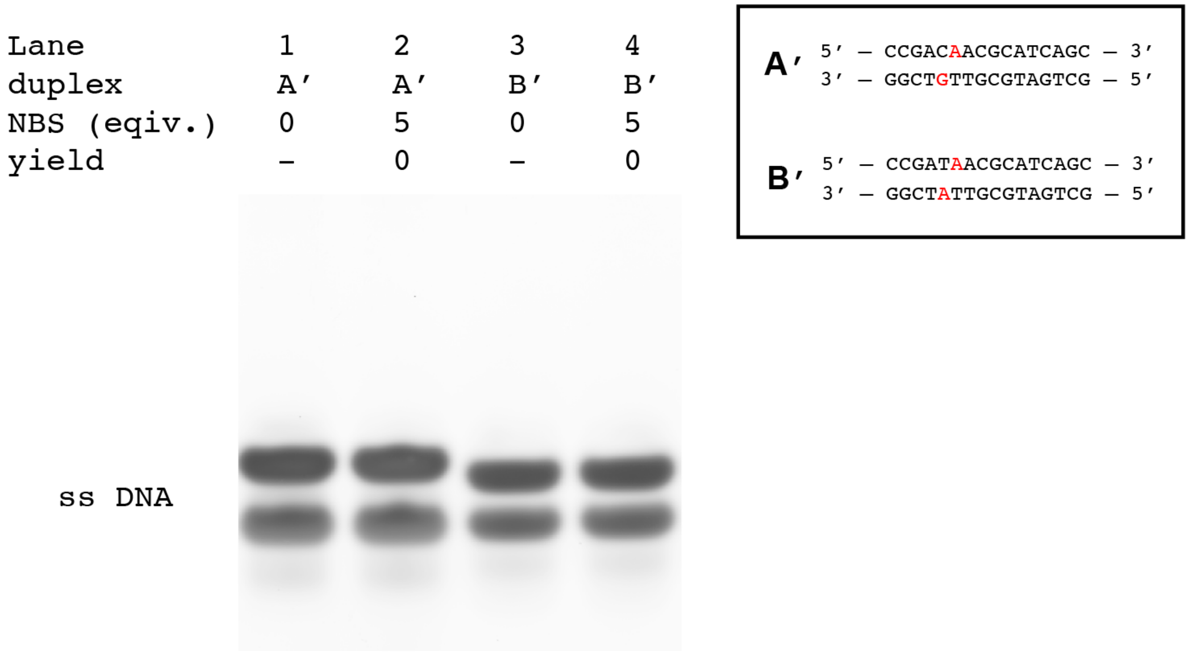

Supplementary Fig. 1: Denaturing urea polyacrylamide gel electrophoresis (PAGE) analysis of NBS-catalyzed ICL reactions with undamaged versions of duplexes **A′** and **B′** that contain normal adenine in place of oxoA. This reaction demonstrates that oxoA is required for ICL formation to occur. Template ODN represents the ODN that would normally contain oxoA, while the complementary ODN is the un-modified complementary strand. All yields and related statistics were derived from independent experiments and reported as the mean value ± SEM in the text based on n = 3 independent replicates. OxoA represents 8-oxoadenine, NBS represents N-bromosuccinimide, ODN represents oligodeoxyribonucleotide, and ICL represents interstrand cross-linking.

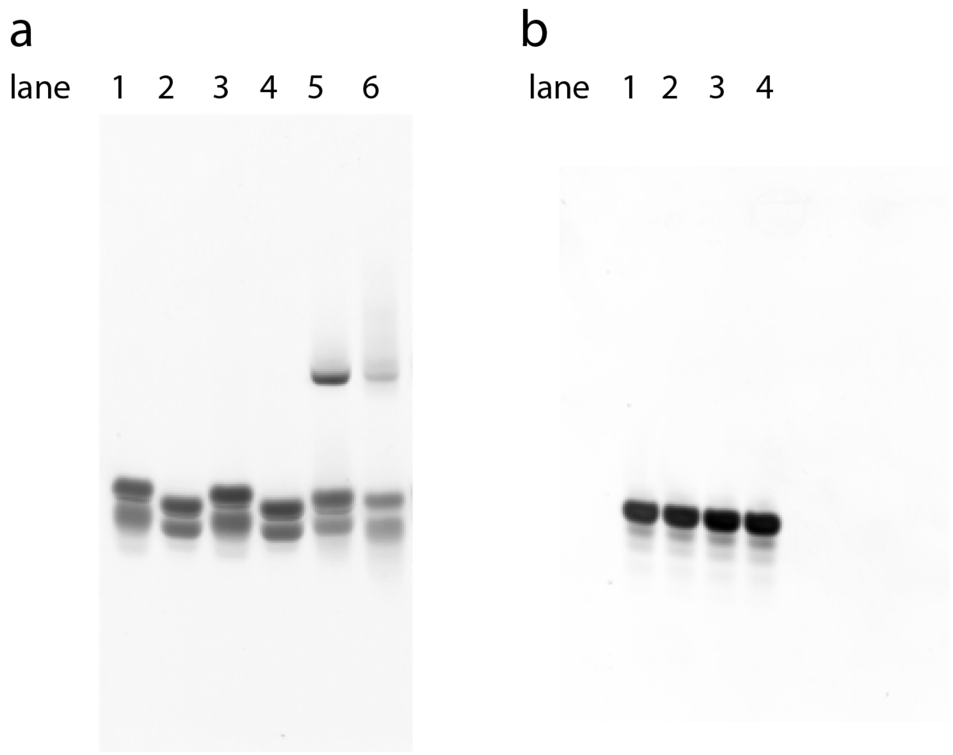

Supplementary Fig. 2: Uncropped and unmodified gel images used to generate Fig. 2a. **a** The gel image contains cross-linked duplex **A** in lane 5 (NBS oxidation) along with a modified version of duplex **A** in lane 6 that contains oxoA base paired with a cytosine residue to compare to the duplex **E** reactions. Associated controls can be found in lanes 1 – 4 (lanes 1 – 2 = no oxoA modification; lanes 3 – 4 = no NBS addition). **b** The gel image contains duplex **E** treated with NBS in lane 4 and without treatment in lane 3. Modified duplex **E** that contains the oxoG modification base paired with a thymine residue to mimic duplex **A** can be found in lanes 1 (no NBS treatment) and 2 (NBS treated). Three independent experiments were conducted to ensure accuracy of reporting.

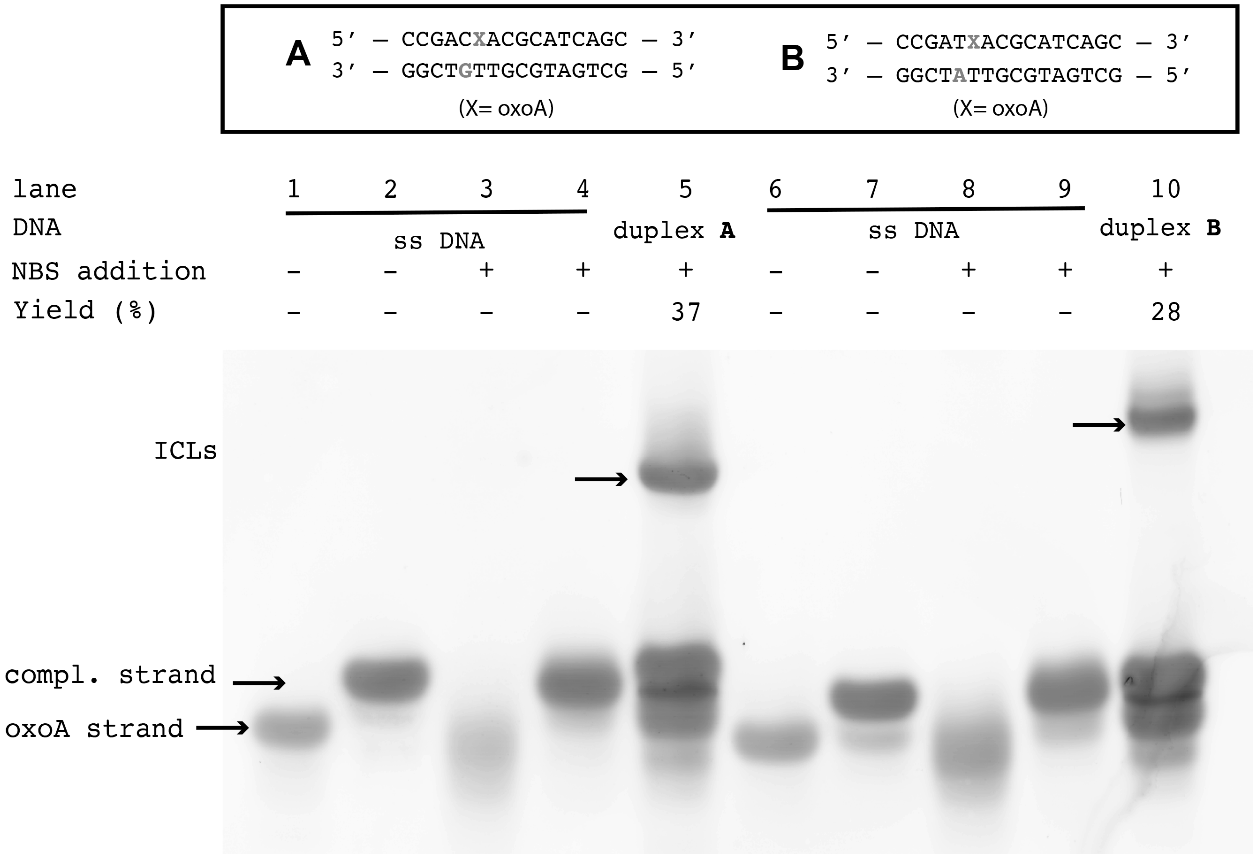

Supplementary Fig. 3: Denaturing urea PAGE analysis of NBS-catalyzed reactions with individual oligonucleotides and dsDNAs from duplexes **A** and **B**. The purpose of these reactions was to demonstrate that cross-links were not observed when individual oligonucleotides were NBS treated. All yields and related statistics were derived from independent experiments and reported as the mean value ± SEM in the text based on n = 3 independent replicates. OxoA represents 8-oxoadenine and is defined as X in the above figure, NBS represents *N*-bromosuccinimide, and ICL represents interstrand cross-linking.

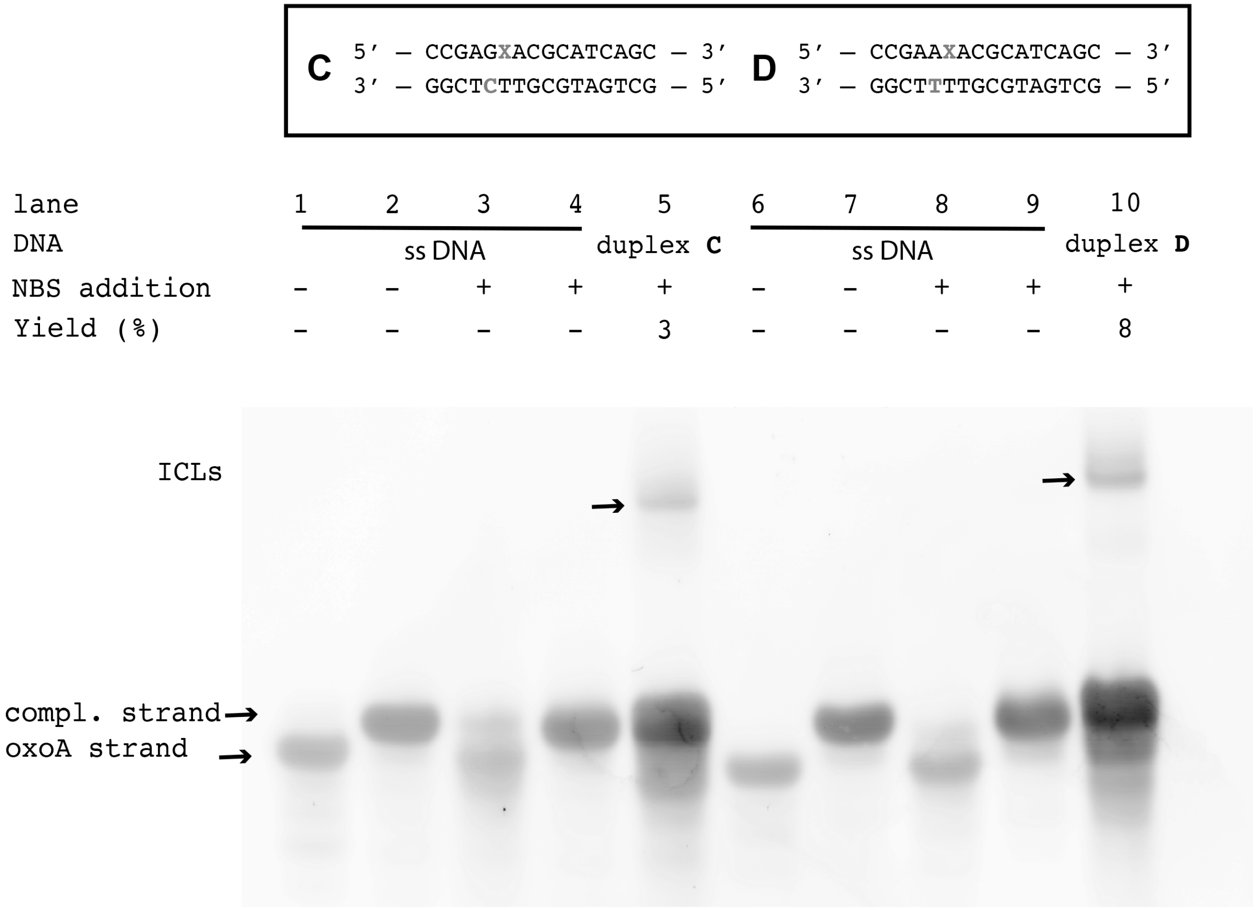

Supplementary Fig. 4: Denaturing urea polyacrylamide gel electrophoresis analysis of NBS-catalyzed reactions with individual oligonucleotides and dsDNAs from duplexes **C** and **D**. The purpose of these reactions was to demonstrate that cross-links were not observed when individual oligonucleotides were NBS treated. All yields and related statistics were derived from independent experiments and reported as the mean value ± SEM in the text based on n = 3 independent replicates. OxoA represents 8-oxoadenine and is defined as X in the above figure, NBS represents N-bromosuccinimide, and ICL represents interstrand cross-linking.

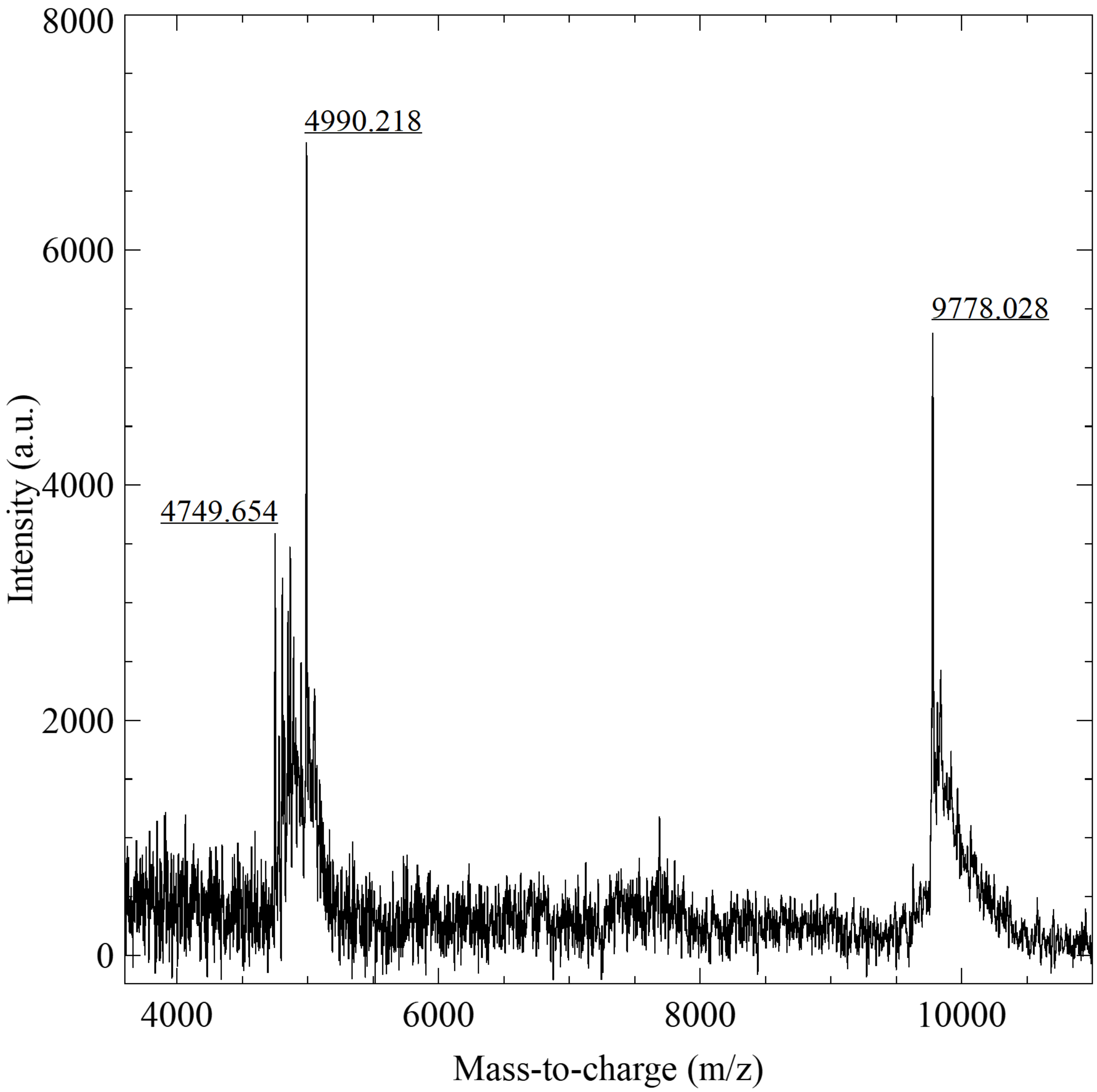

Supplementary Fig. 5: MALDI-TOF mass spectrometric analysis of cross-linked duplex **A**, which represents the oxoA-G ICL. Calculated mass = 9778.446. The cross-linked DNA was PAGE purified, ethanol precipitated, and desalted with C4-resin ZipTips. The spectrum was externally calibrated with a range of ODN standards (6046 Da – 12013 Da).

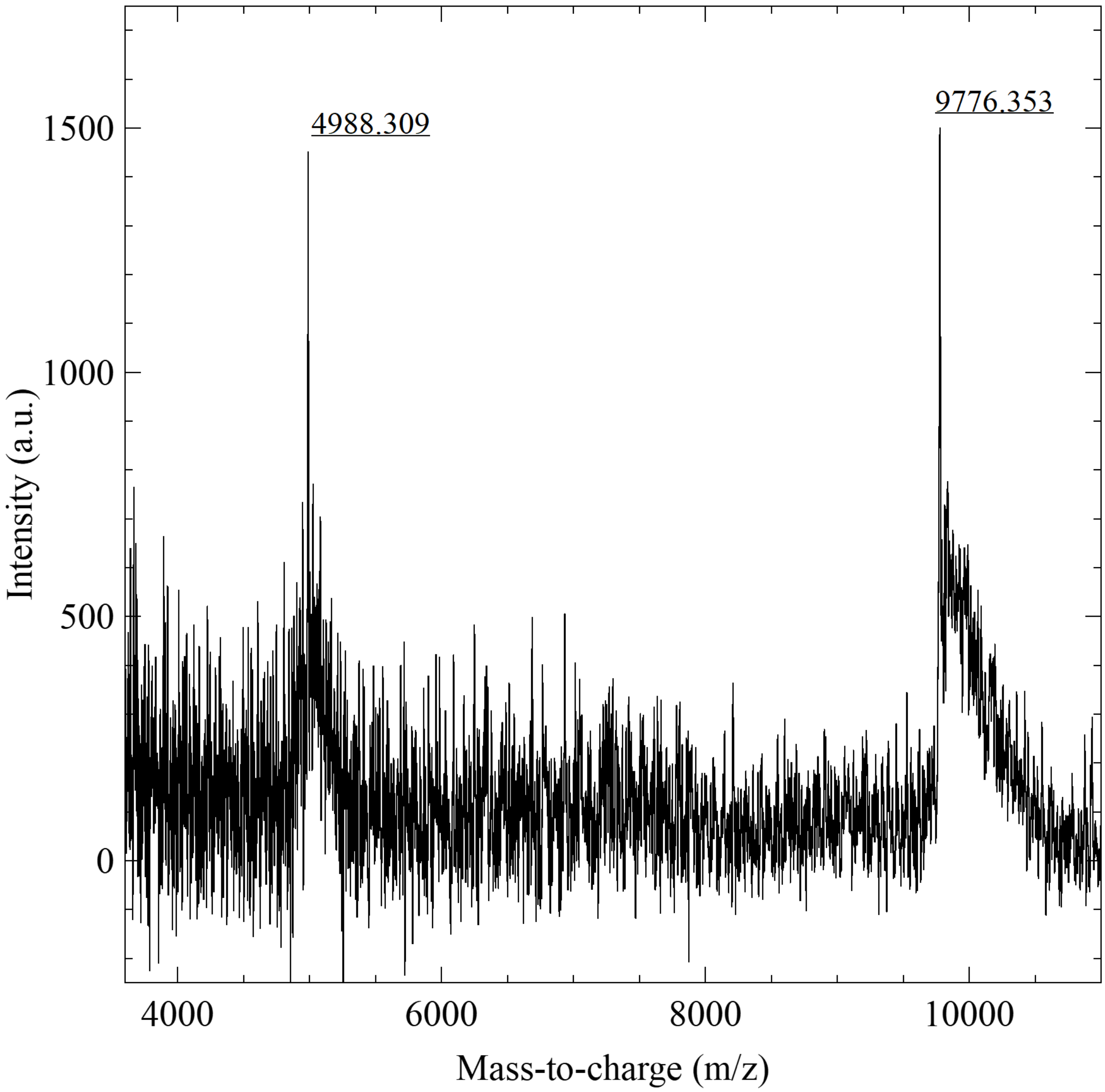

Supplementary Fig. 6: MALDI-TOF mass spectrometric analysis of cross-linked duplex **B**, which represents the oxoA-A ICL. Calculated mass = 9778.466. The cross-linked DNA was PAGE purified, ethanol precipitated, and desalted with C4-resin ZipTips. The spectrum was externally calibrated with a range of ODN standards (6046 Da – 12013 Da).

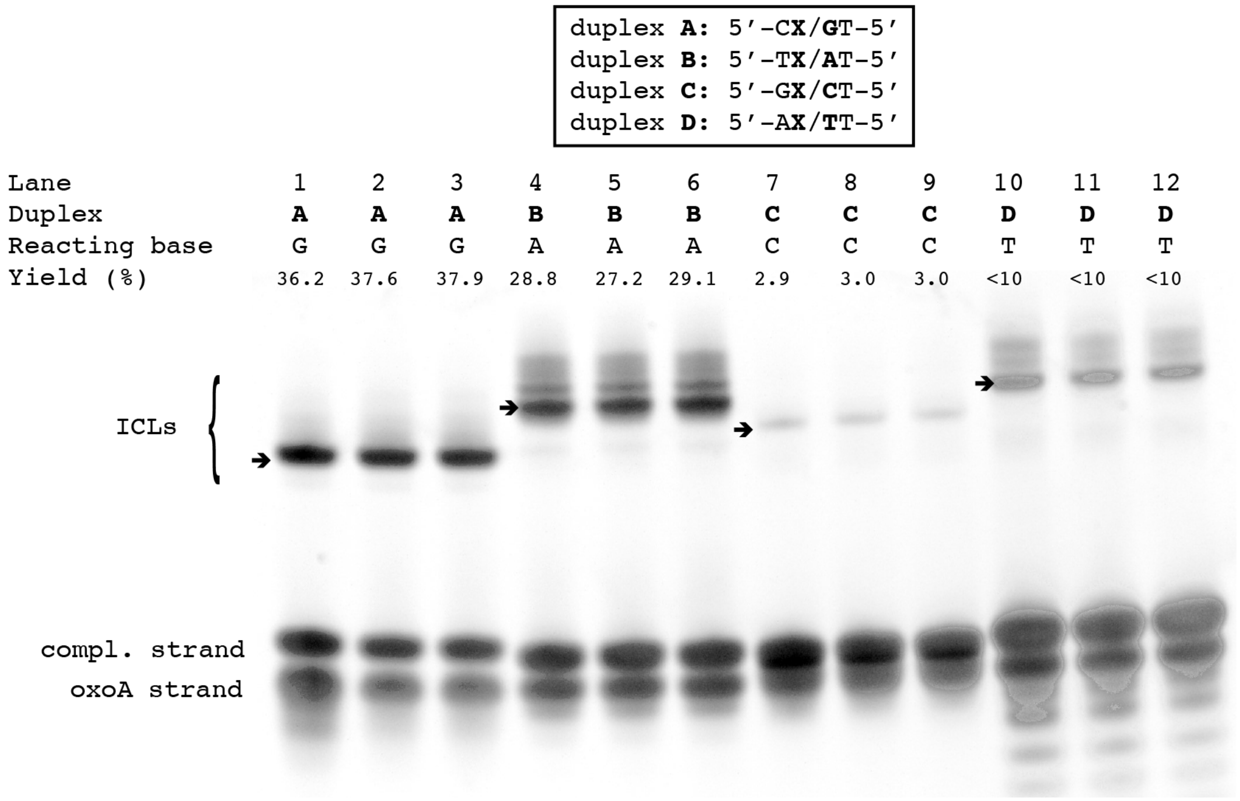

Supplementary Fig. 7: Cross-link formation between oxoA and an opposite nucleobase in duplex DNA. Denaturing urea polyacrylamide gel electrophoresis analysis of triplicate NBS-catalyzed ICL reactions with duplexes **A**-**D**. The oxoA strand represents the oxoA-modified strand, while the complementary strand is the un-modified complementary strand. The oxoA ICLs used for ICL yield calculation are indicated as arrows. Cross-linking yields were determined by dividing the percent band intensity of the cross-linked DNA band by the sum of the intensities of the unreacted complementary and oxoA strands. OxoA represents 8-oxoadenine and is defined as X in the above figure, NBS represents N-bromosuccinimide, and ICL represents interstrand cross-linking.

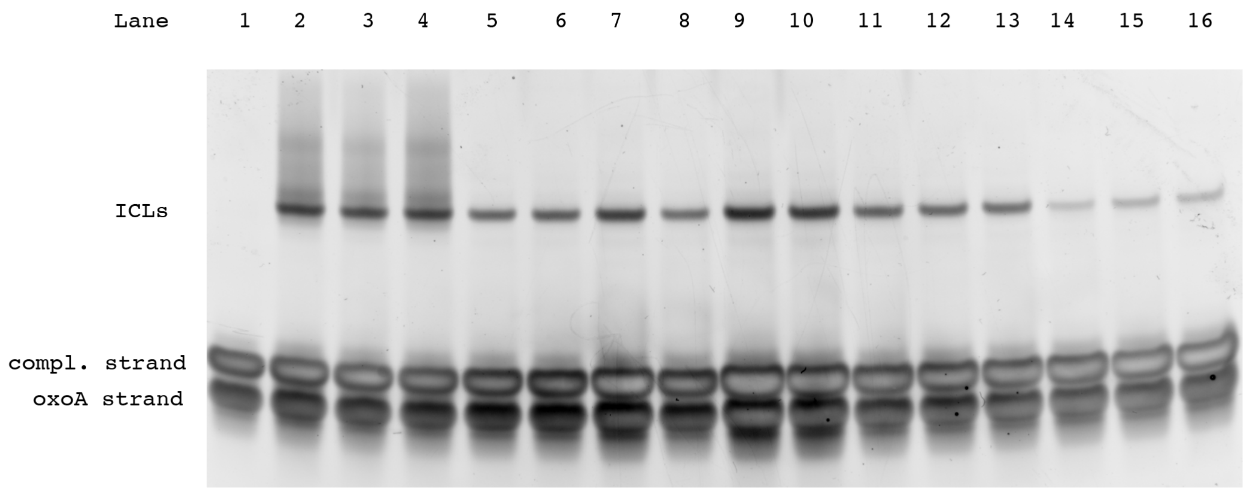

	MPO (nM)	oxidant	Yield (%)
1	-	-	0
2	-	NaOCl (100 molar eq.)	23.1
3	-	NaOCl (100 molar eq.)	21.9
4	-	NaOCl (100 molar eq.)	24.0
5	50	H_2O_2 (250 μM)	9.9
6	50	H_2O_2 (250 μM)	10.7
7	50	H_2O_2 (250 μM)	9.5
8	150	H_2O_2 (250 μM)	14.5
9	150	H_2O_2 (250 μM)	17.9
10	150	H_2O_2 (250 μM)	16.7
11	325	H_2O_2 (250 μM)	14.1
12	325	H_2O_2 (250 μM)	12.7
13	325	H_2O_2 (250 μM)	12.6
14	500	H_2O_2 (250 μM)	3.5
15	500	H_2O_2 (250 μM)	5.5
16	500	H_2O_2 (250 μM)	5.8

Supplementary Fig. 8: Denaturing urea polyacrylamide gel electrophoresis analysis of triplicate MPO-H_2O_2-Cl-catalyzed ICL reactions with duplex **A** at various conditions. Duplex A contains 5´-CoxoA/GT-5´ base pairs. Cross-linking yields were determined by dividing the percent band intensity of the cross-linked DNA band by the sum of the intensities of the unreacted complementary and oxoA strands. Detailed reaction conditions are shown in the table.

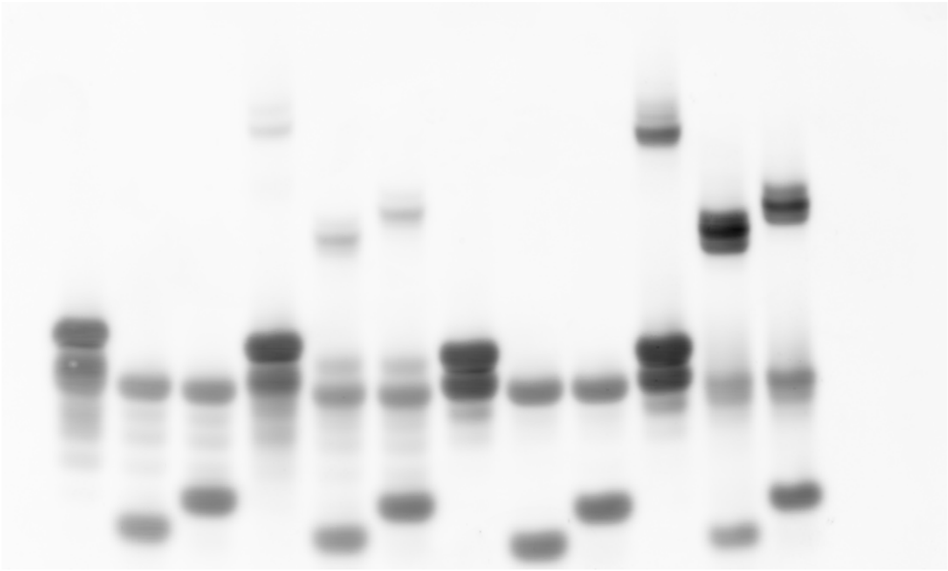

Supplementary Fig. 9: Full, uncropped source gel image for Fig. 8. Denaturing urea PAGE analysis of cross-linking reactions involving duplexes **M** – **P**. Note that lanes 6 and 12 contain cross-linking reactions involving modified versions of duplexes **P** and **O**, respectively. These duplexes contain 5’ overhangs on the complementary strands that were intended to prevent further melting but were determined unnecessary. Furthermore, the yields for these two cross-links were greater than the unresected duplexes and virtually identical to duplexes **P** and **O**.

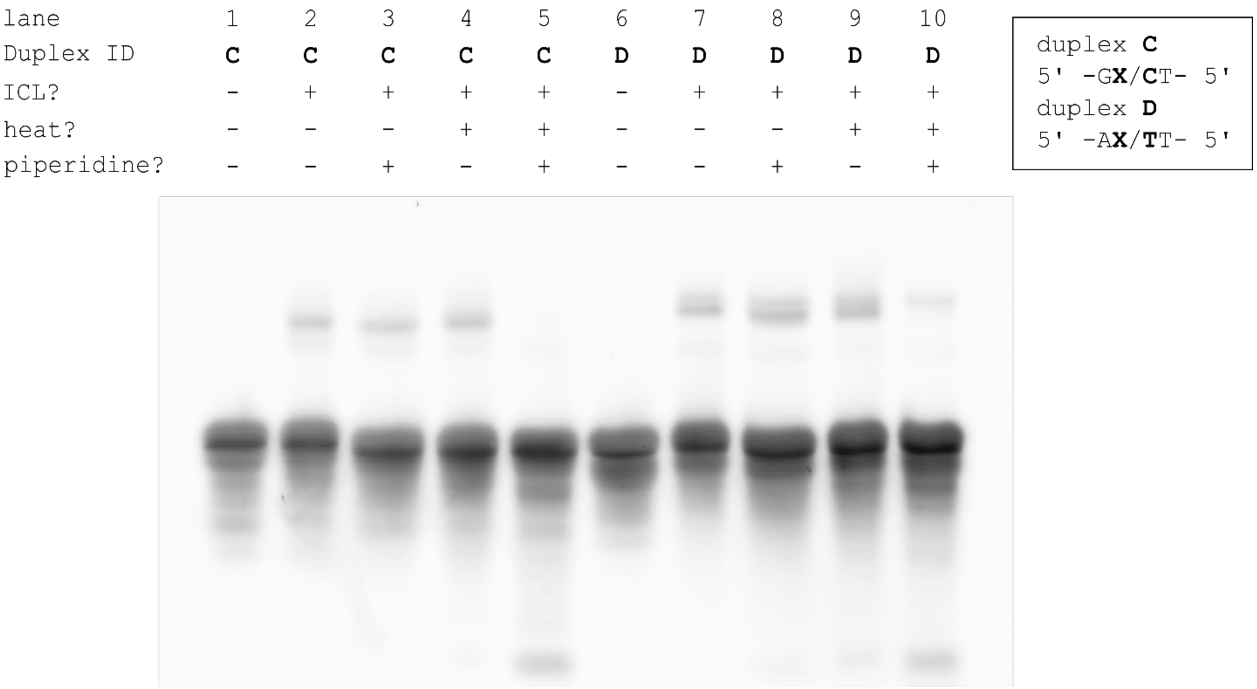

Supplementary Fig. 10: Denaturing urea PAGE analysis of cross-linked duplexes C and D treated with various combinations of heat and/or piperidine. Duplexes **C** and **D** contain 5´-GoxoA/CT-5´ and 5´-AoxoA/TT-5´sequence, respectively, where nucleophilic bases are underlined. The duplex **C** and **D** cross-linking reactions were heated for 30 mins at 90 °C in the presence/absence of piperidine. The concentrated samples were resuspended in the formamide loading buffer and subjected to PAGE analysis. OxoA represents 8-oxoadenine and is defined as X in the above figure and ICL represents interstrand cross-linking.

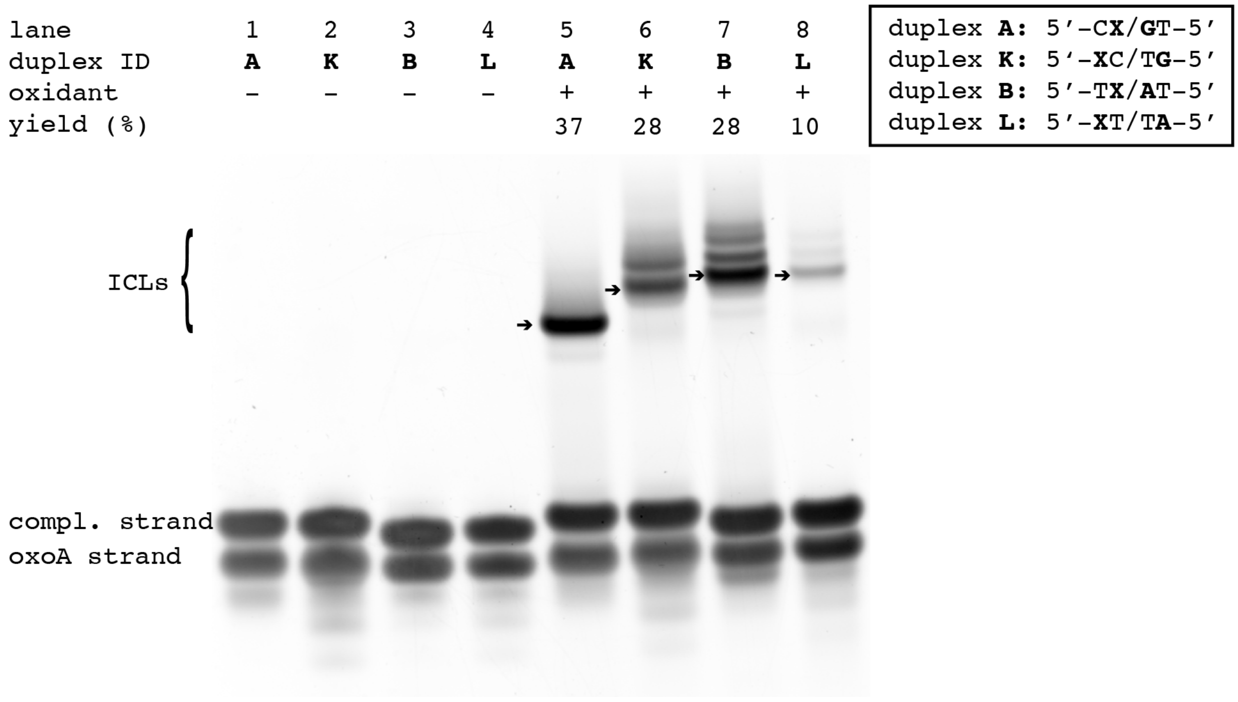

Supplementary Fig. 11: The effect of sequence on the oxoA cross-linking reaction. A denaturing urea PAGE analysis of NBS-catalyzed oxoA cross-linking is shown. Slow-migrating bands are oxoA ICLs. The major ICLs from individual reactions are indicated as arrows and their ICL yields are shown. Cross-linking yields were determined by dividing the percent band intensity of the cross-linked DNA band by the sum of the intensities of the unreacted complementary and oxoA strands. Yields are averages of three independent determinations. Duplexes contain 5′-CoxoA/GT-5′ (A), 5′-oxoAC/TG-5′ (K), 5′-ToxoA/AT-5′ (B), and 5′-oxoAT/TA-5′ (L) sequences, where nucleophilic bases are underlined. All yields and related statistics were derived from independent experiments and reported as the mean value ± SEM in the text based on n = 3 independent replicates. OxoA represents 8-oxoadenine and is defined as X in the above figure, NBS represents *N*-bromosuccinimide, and ICL represents interstrand cross-linking.

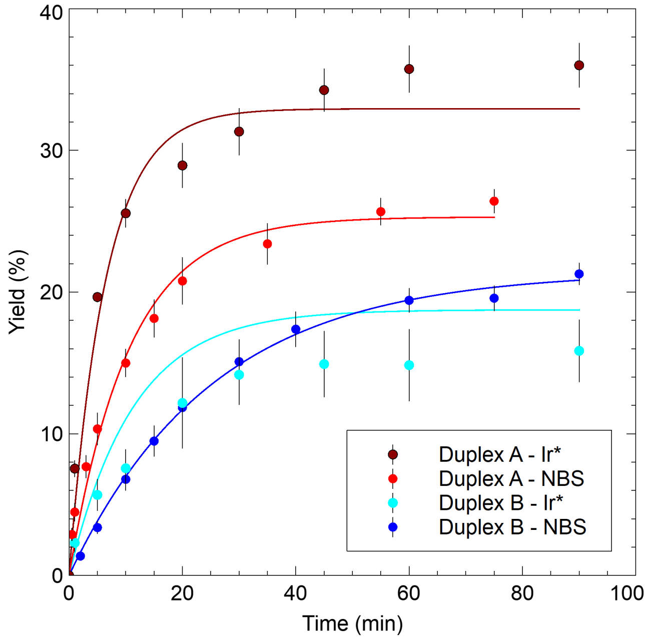

**Reaction ID**	duplex **A** – NBS	duplex **A** – Ir	duplex **B** – NBS	duplex **B** – Ir
k_{ICL} **(x 10^3 s^{-1})**	1.6 ± 0.3	2.6 ± 0.1	0.7 ± 0.1	1.5 ± 0.2
$t_{1/2}$ **(min)**	7.7 ± 1.4	4.5 ± 0.4	18.8 ± 3.1	7.5 ± 1.2

Supplementary Fig. 12: Time-course analysis of the major oxoA cross-linking reactions with NBS and Na_2IrCl_6 oxidation. Kinetic plot displays observed ICL yields for oxidation reactions determined from denaturing PAGE analysis over time. Curves were fit to $Y = ICL_{MAX}*(1 - \exp[-k_{ICL}*t])$ (1), where ICL_{MAX} corresponds to the maximum observed ICL yield. Cross-linking yields were determined by dividing the percent band intensity of the cross-linked DNA band by the sum of the intensities of the unreacted complementary and oxoA strands from PAGE analysis. Kinetic parameters determined from non-linear regression analysis of the yield data where k_{ICL} is the rate constant for the reaction and $t_{1/2}$ is defined as the reaction half-life. All yields, related kinetic values and statistics were derived from independent experiments and reported as the mean value ± SEM based on n = 3 independent replicates. OxoA represents 8-oxoadenine, NBS represents *N*-bromosuccinimide, Ir* represents Na_2IrCl_6, and ICL represents interstrand cross-linking.

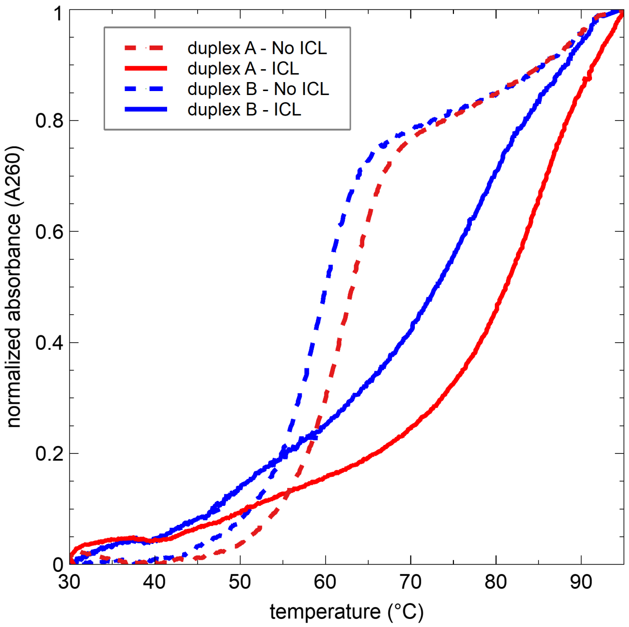

Supplementary Fig. 13: Thermal stability curves and melting temperatures for cross-linked and non-cross-linked duplexes A and B. Dotted curves represent data for non-cross-linked duplexes, whereas solid lines represent data for cross-linked duplexes. Melting curves were monitored at 260 nm from 30°C to 95 °C with a temperature gradient of 1.0 °C/min and performed in triplicate to determine melting temperatures. The steep increase in absorbance with temperature in the cross-linked duplexes is characteristic of ICL-bearing DNA, as the covalent linkage strengthens hydrogen bonding interactions near the cross-link. ICL represents interstrand cross-linking.

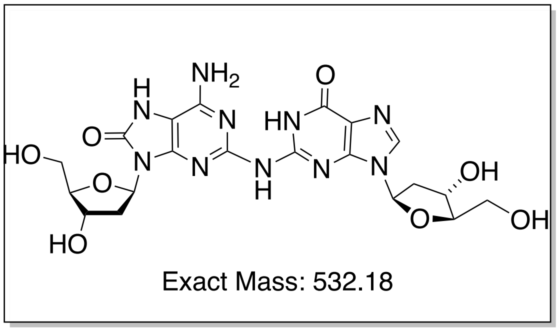

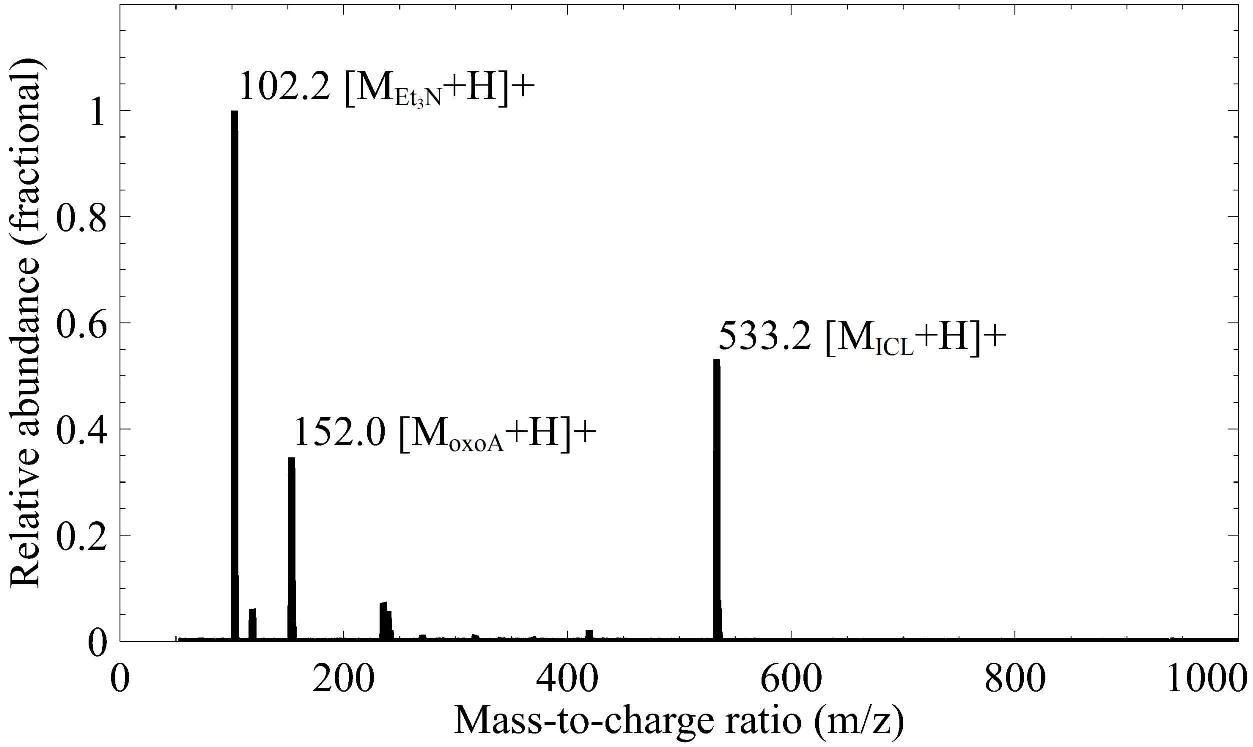

Supplementary Fig. 14: Full mass signal (MS) from Fig. 9a demonstrating an oxoA-G cross-linked dinucleoside. Adducts are labeled for peaks that were able to be defined. Note that triethylamine (Et_3N) arises from HPLC mobile phase (0.1 M triethylammonium acetate).

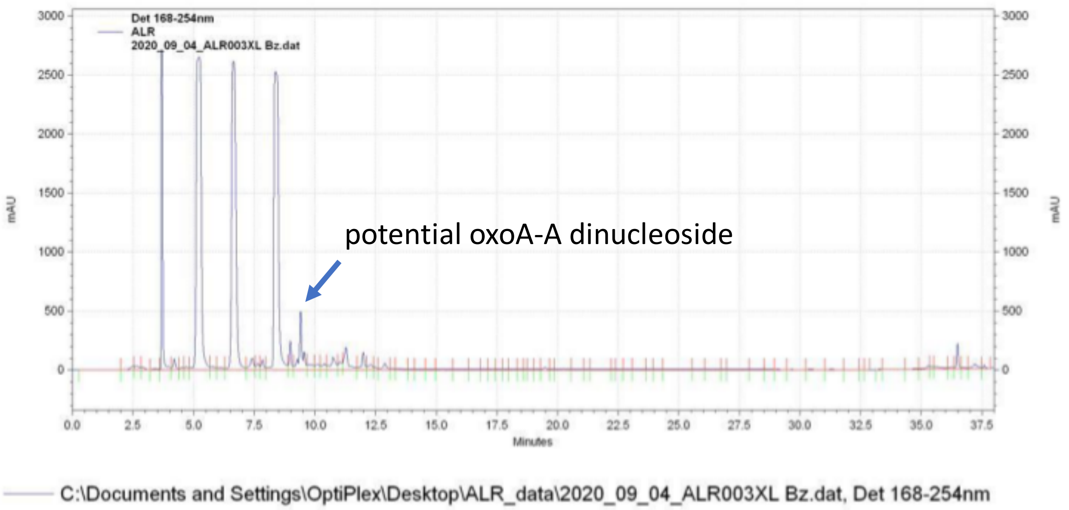

Supplementary Fig. 15: HPLC chromatogram derived from purification of cross-linked duplex B enzymatic digestion mixtures. Peaks for individual nucleosides were determined as follows: deoxycytidine at 3.52 min, deoxyguanosine at 5.15 min, deoxythymidine at 6.75 min, and deoxyadenosine at 8.5 min. Peak at 9.5 min was believed to be the major oxoA-A cross-linked dinucleoside and represents the peak subjected to LC-MS analysis in Supplementary Fig. 16. Minor peaks at 9 min, 10.75 min, and 12 min were not able to be determined by LC-MS analysis. Interestingly, the peak at 12 min may represent a cross-linked oxoA-T dinucleoside given the similar retention time.

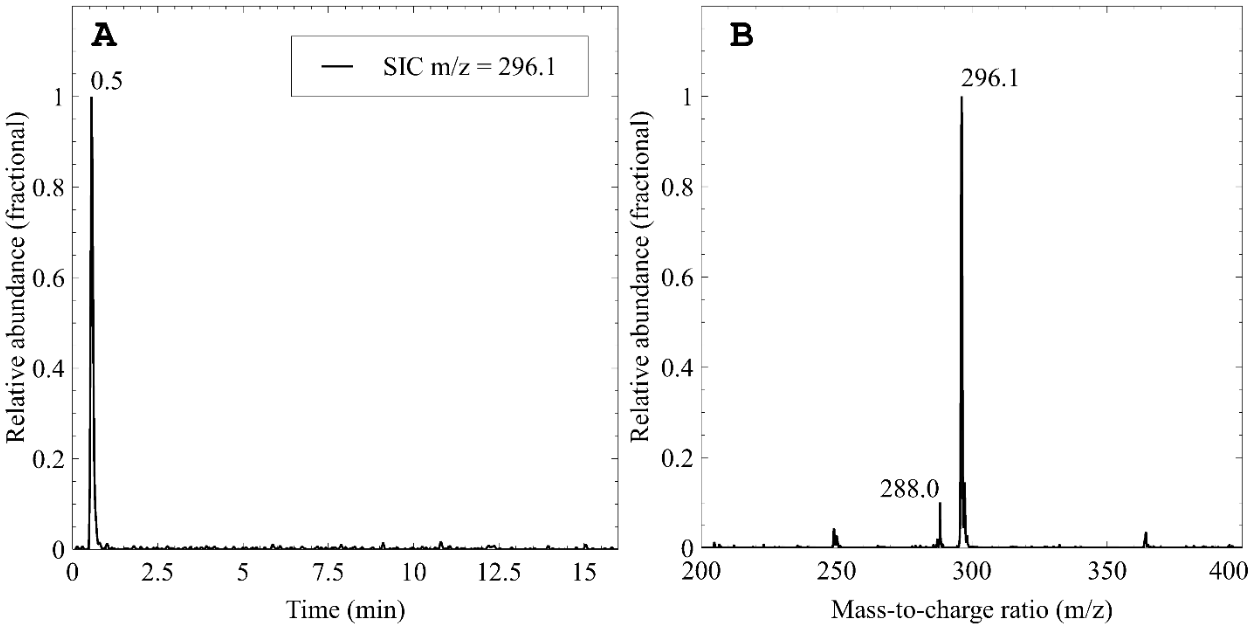

Supplementary Fig. 16: Selected-ion chromatogram (SIC, A) and mass signal (B) for peak eluting at 0.5 min arising from concentrated HPLC fraction believed to contain oxoA-A cross-linked dinucleoside. Signal corresponding to an oxoA nucleoside is observed at *m/z* 288.1 ([M+Na-2H]$^-$). Signal corresponding to an adenine nucleoside is observed at *m/z* 296.1 ([M+HCOOH-H]-). This provides evidence that the cross-link formed upon oxidation of duplex **B** is an oxoA-A cross-link.

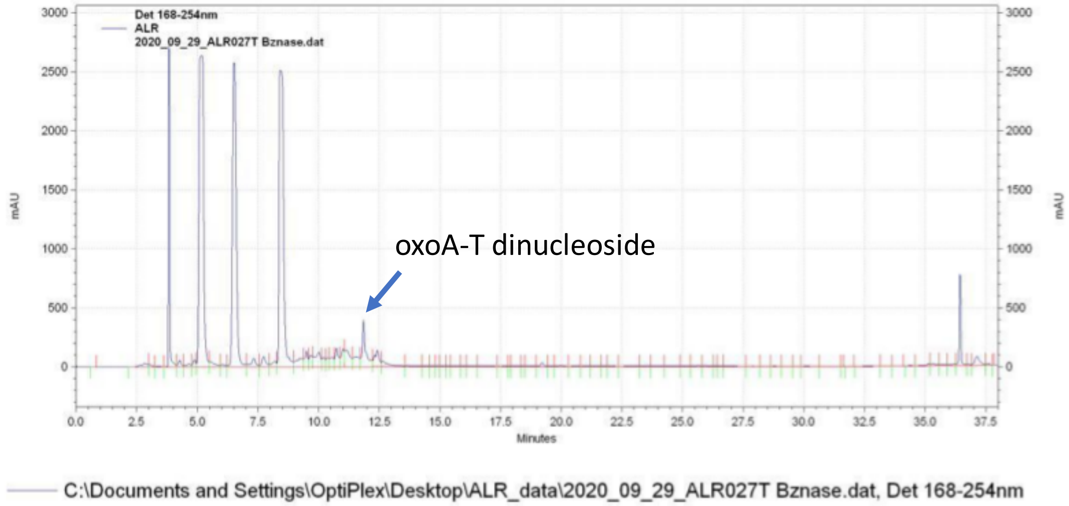

Supplementary Fig. 17: HPLC chromatogram derived from purification of cross-linked duplex D enzymatic digestion mixtures. Peaks for individual nucleosides were determined as follows: deoxycytidine at 3.52 min, deoxyguanosine at 5.15 min, deoxythymidine at 6.75 min, and deoxyadenosine at 8.5 min. Peak at 12 min was determined to be the cross-linked oxoA-T dinucleoside by LC-MS analysis (**Fig. 9b**). No other major peaks were observed, which suggests that the only cross-links generated by oxidation of duplex **D** occur between oxoA and thymine residues.

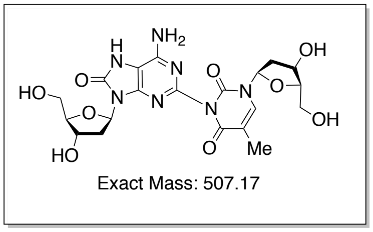

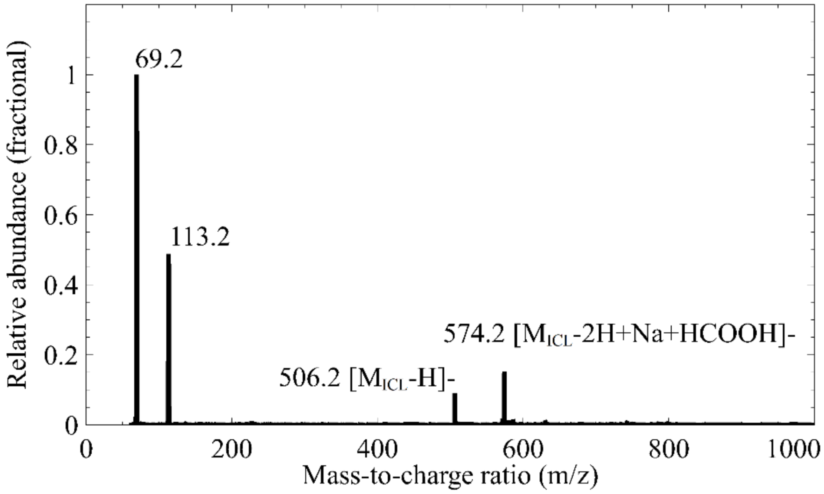

Supplementary Fig. 18: Full mass signal (MS) from Fig. 9b demonstrating an oxoA-T cross-linked dinucleoside. Adducts are labeled for peaks that were able to be defined. Signals at 69.2 and 113.2 were not able to be defined.

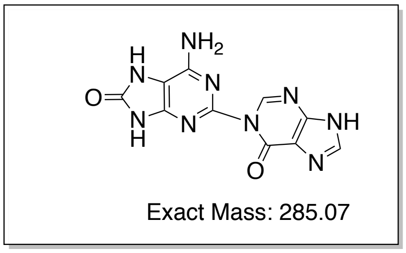

Possible structure of the oxoA-I covalent adduct

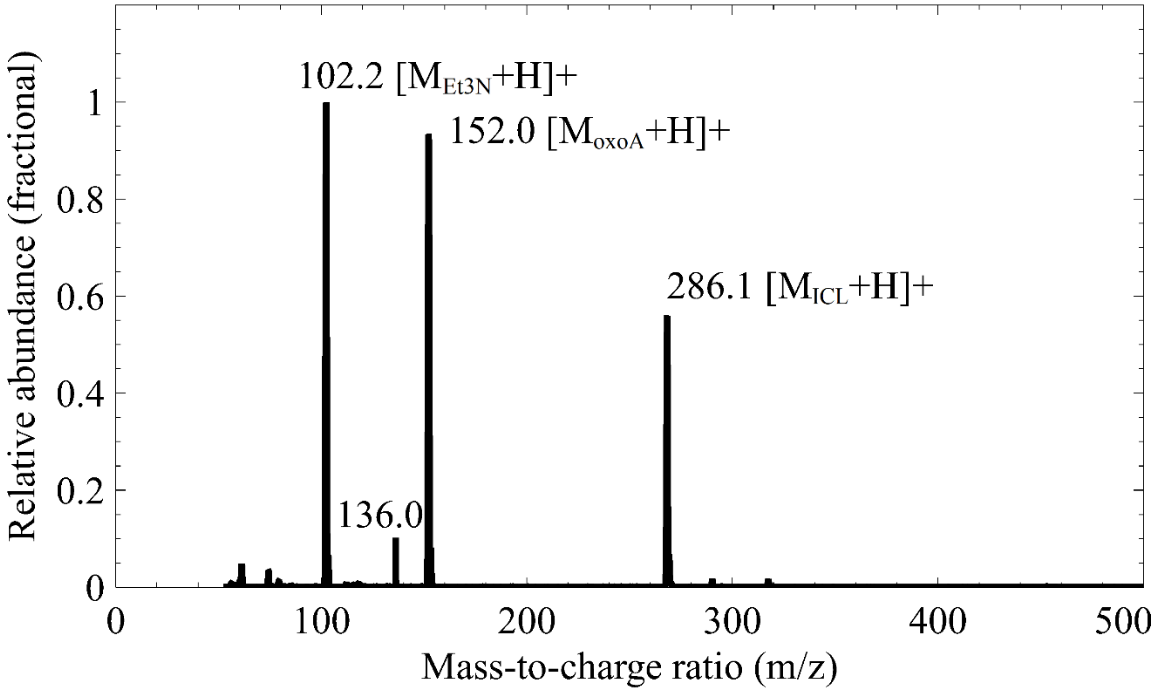

Supplementary Fig. 19: Full mass signal (MS) from Fig. 9c demonstrating an oxoA-I cross-linked dinucleoside. Adducts are labeled for peaks that were able to be defined. Signal at *m/z* 286.1 represents cross-linked oxoA and hypoxanthine purine bases in the form of a $[M_{ICL}+H]+$ adduct. Signal at *m/z* 136.0 represents a hypoxanthine $[M_{HX}+H]+$ adduct. Note that triethylamine (Et_3N) arises from HPLC mobile phase (0.1 M triethylammonium acetate).
