# Supplementary material for: DNA interstrand cross-links induced by the major oxidative adenine lesion 7,8-dihydro-8-oxoadenine
Source: Nat Commun. 2021 Mar 26;12:1897. doi: 10.1038/s41467-021-22273-2 (PMC7997976; doi:10.1038/s41467-021-22273-2)
Supplement: Supplementary file 2 — Reporting Summary [file 41467_2021_22273_MOESM2_ESM.pdf]

nature research

Corresponding author(s): Seongmin Lee

Last updated by author(s): Dec 21, 2020

Reporting Summary

Nature Research wishes to improve the reproducibility of the work that we publish. This form provides structure for consistency and transparency in reporting. For further information on Nature Research policies, see our Editorial Policies and the Editorial Policy Checklist.

Statistics

For all statistical analyses, confirm that the following items are present in the figure legend, table legend, main text, or Methods section.

n/a	Confirmed	
☐	☒	The exact sample size (n) for each experimental group/condition, given as a discrete number and unit of measurement
☐	☒	A statement on whether measurements were taken from distinct samples or whether the same sample was measured repeatedly
☒	☐	The statistical test(s) used AND whether they are one- or two-sided<br>*Only common tests should be described solely by name; describe more complex techniques in the Methods section.*
☐	☒	A description of all covariates tested
☒	☐	A description of any assumptions or corrections, such as tests of normality and adjustment for multiple comparisons
☐	☒	A full description of the statistical parameters including central tendency (e.g. means) or other basic estimates (e.g. regression coefficient) AND variation (e.g. standard deviation) or associated estimates of uncertainty (e.g. confidence intervals)
☒	☐	For null hypothesis testing, the test statistic (e.g. F, t, r) with confidence intervals, effect sizes, degrees of freedom and P value noted<br>*Give P values as exact values whenever suitable.*
☒	☐	For Bayesian analysis, information on the choice of priors and Markov chain Monte Carlo settings
☒	☐	For hierarchical and complex designs, identification of the appropriate level for tests and full reporting of outcomes
☒	☐	Estimates of effect sizes (e.g. Cohen's d, Pearson's r), indicating how they were calculated

Our web collection on statistics for biologists contains articles on many of the points above.

Software and code

Policy information about availability of computer code

Data collection	GE Typhoon FLA 9500 Control Software (version unspecified), Thermo INSIGHT analysis software (V 2.4), Bruker flexAnalysis software (version unspecified), Agilent ChemStation (V B.04.03)
Data analysis	Data analysis is described in the Methods section. The following software were used for all analysis: GE ImageQuant TL (V 8.1.0) for quantifying PAGE analysis, UCSF Chimera (V 1.12) used in conjunction with PyMOL to generate molecular models, Thermo INSIGHT analysis software (V 2.4) for determining melting temperatures of duplex DNAs, PyMOL (V 2.02), to generate molecular models, Veusz (V 3.3.1) for generating plots, Microsoft Excel for statistical analyses of reaction yields and kinetics, Bruker flexAnalysis software (version unspecified) for analyzing MALDI-TOF-MS data, Agilent ChemStation (V B.04.03) for analyzing LC-MS data.

For manuscripts utilizing custom algorithms or software that are central to the research but not yet described in published literature, software must be made available to editors and reviewers. We strongly encourage code deposition in a community repository (e.g. GitHub). See the Nature Research guidelines for submitting code & software for further information.

Data

Policy information about availability of data

All manuscripts must include a data availability statement. This statement should provide the following information, where applicable:

- Accession codes, unique identifiers, or web links for publicly available datasets
- A list of figures that have associated raw data
- A description of any restrictions on data availability

Molecular models generated in this manuscript are based on the following PDB files: 1BNA, 1S9O.
The datasets generated during and/or analysed during the current study are available in the Source Data File included with this manuscript. Further data may be supplied by the corresponding author upon reasonable request.

Field-specific reporting

Please select the one below that is the best fit for your research. If you are not sure, read the appropriate sections before making your selection.

- [x] Life sciences
- [ ] Behavioural & social sciences
- [ ] Ecological, evolutionary & environmental sciences

For a reference copy of the document with all sections, see nature.com/documents/nr-reporting-summary-flat.pdf

Life sciences study design

All studies must disclose on these points even when the disclosure is negative.

Sample size	Unless otherwise stated in the manuscript, all relevant experimental values (e.g., yields, melting temperature, etc.) were determined from three independent experiments and reported as mean +/- SEM.
Data exclusions	No data were excluded from this report
Replication	All data related to reaction yields were confirmed through three independent experiments. All mass spectrometry experiments were repeated twice to confirm validity of data. All thermal stability experiments were repeated three times to confirm validity of data.
Randomization	Randomization was not relevant to this study. All control groups in this study were negative controls of the same type of sample; that is, only one variable was in need of control, so randomization was not necessary.
Blinding	Blinding was not relevant to this study. Similarly to above, only one variable was being controlled in the various experiments in this study, so blinding was unnecessary.

Reporting for specific materials, systems and methods

We require information from authors about some types of materials, experimental systems and methods used in many studies. Here, indicate whether each material, system or method listed is relevant to your study. If you are not sure if a list item applies to your research, read the appropriate section before selecting a response.

Materials & experimental systems

n/a	Involved in the study	
x		Antibodies
x		Eukaryotic cell lines
x		Palaeontology and archaeology
x		Animals and other organisms
x		Human research participants
x		Clinical data
x		Dual use research of concern

Methods

n/a	Involved in the study	
x		ChIP-seq
x		Flow cytometry
x		MRI-based neuroimaging
